# Supplementary figures and images for: McsB forms a gated kinase chamber to mark aberrant bacterial proteins for degradation
Source: eLife. 2021 Jul 30;10:e63505. doi: 10.7554/eLife.63505 (PMC8370763; doi:10.7554/eLife.63505)

**Figure 1c**

Black Boxes indicate the regions shown in the Figure


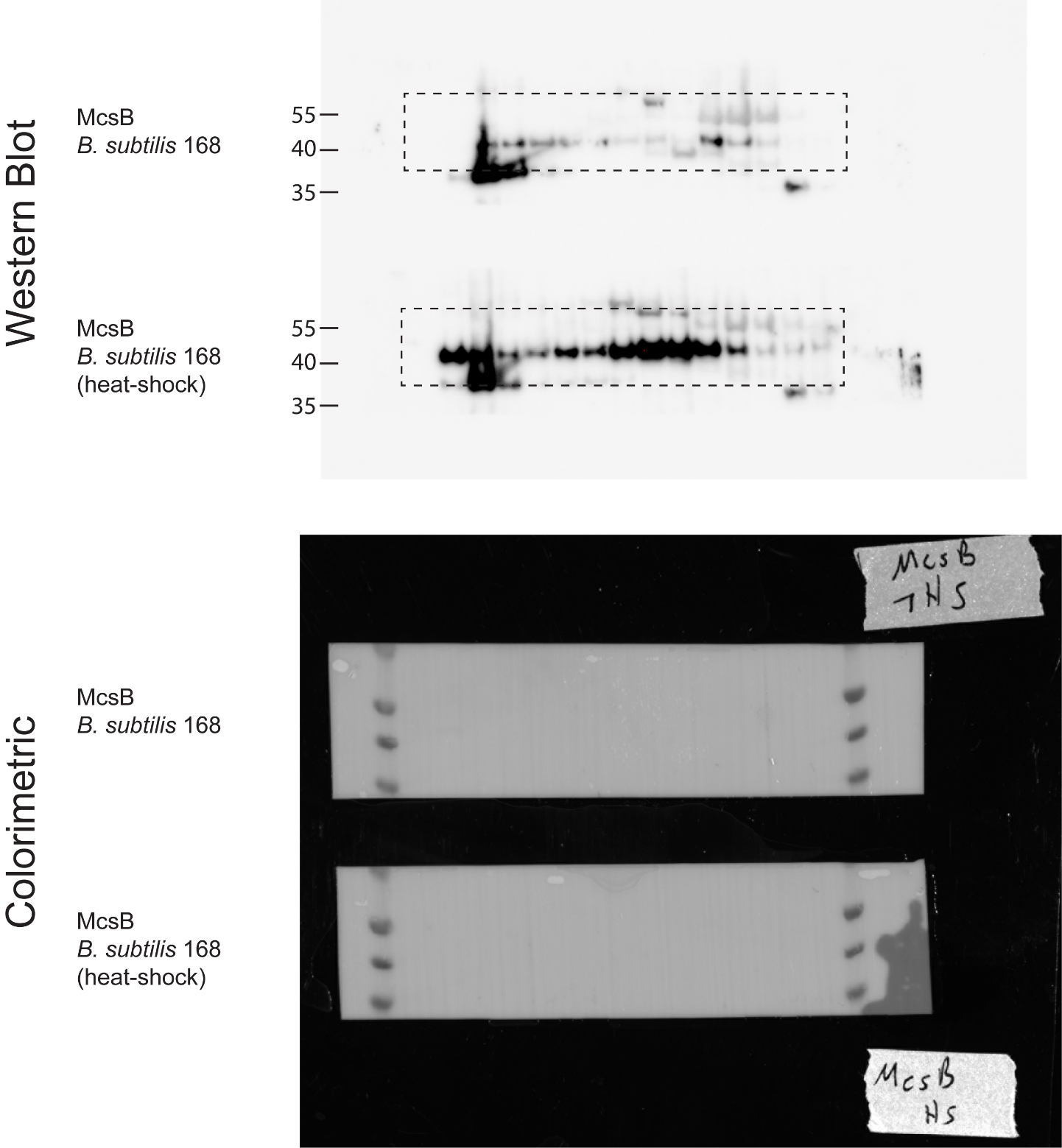


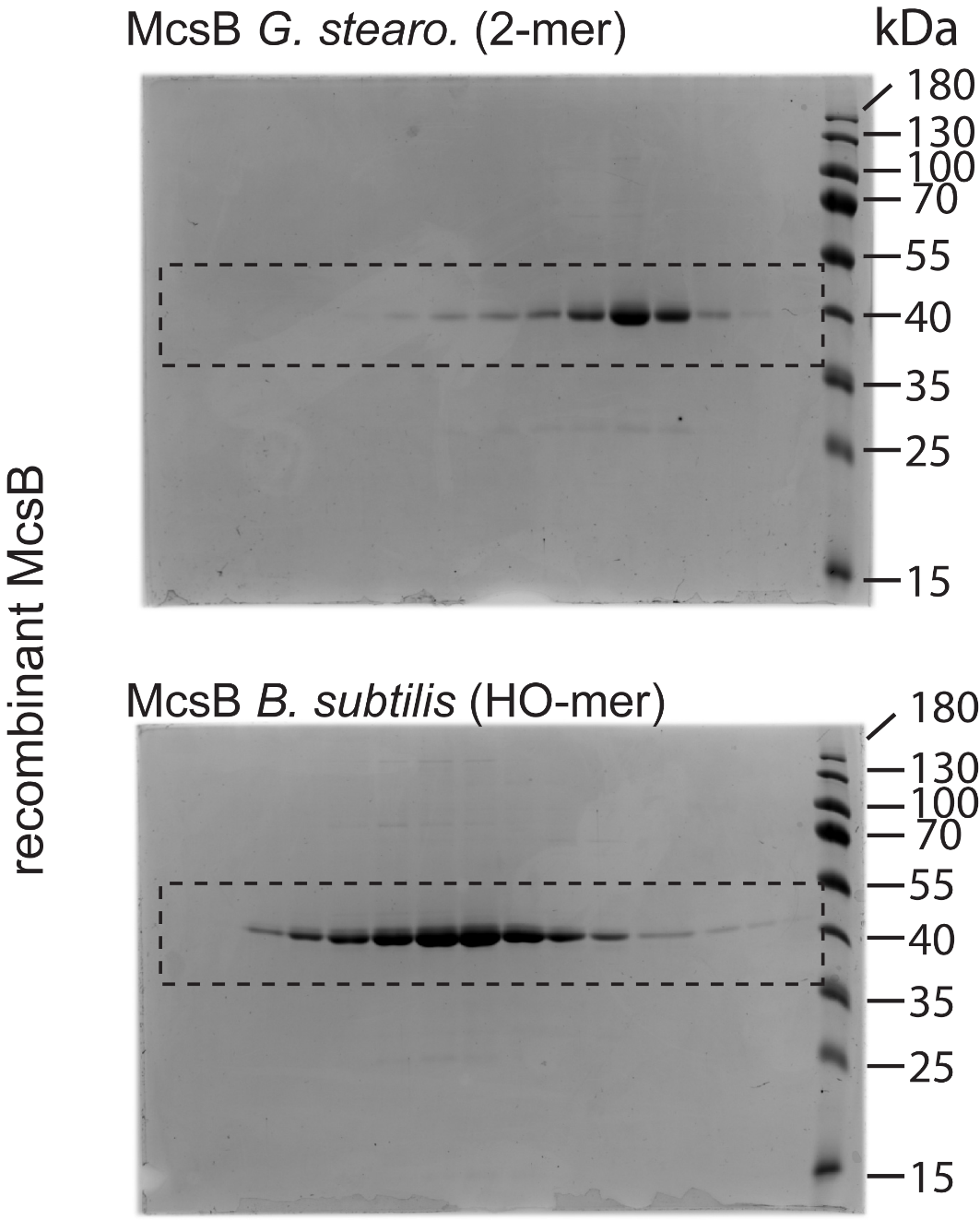

Supplement: Figure 1—source data 1. [file elife-63505-fig1-data1.docx]

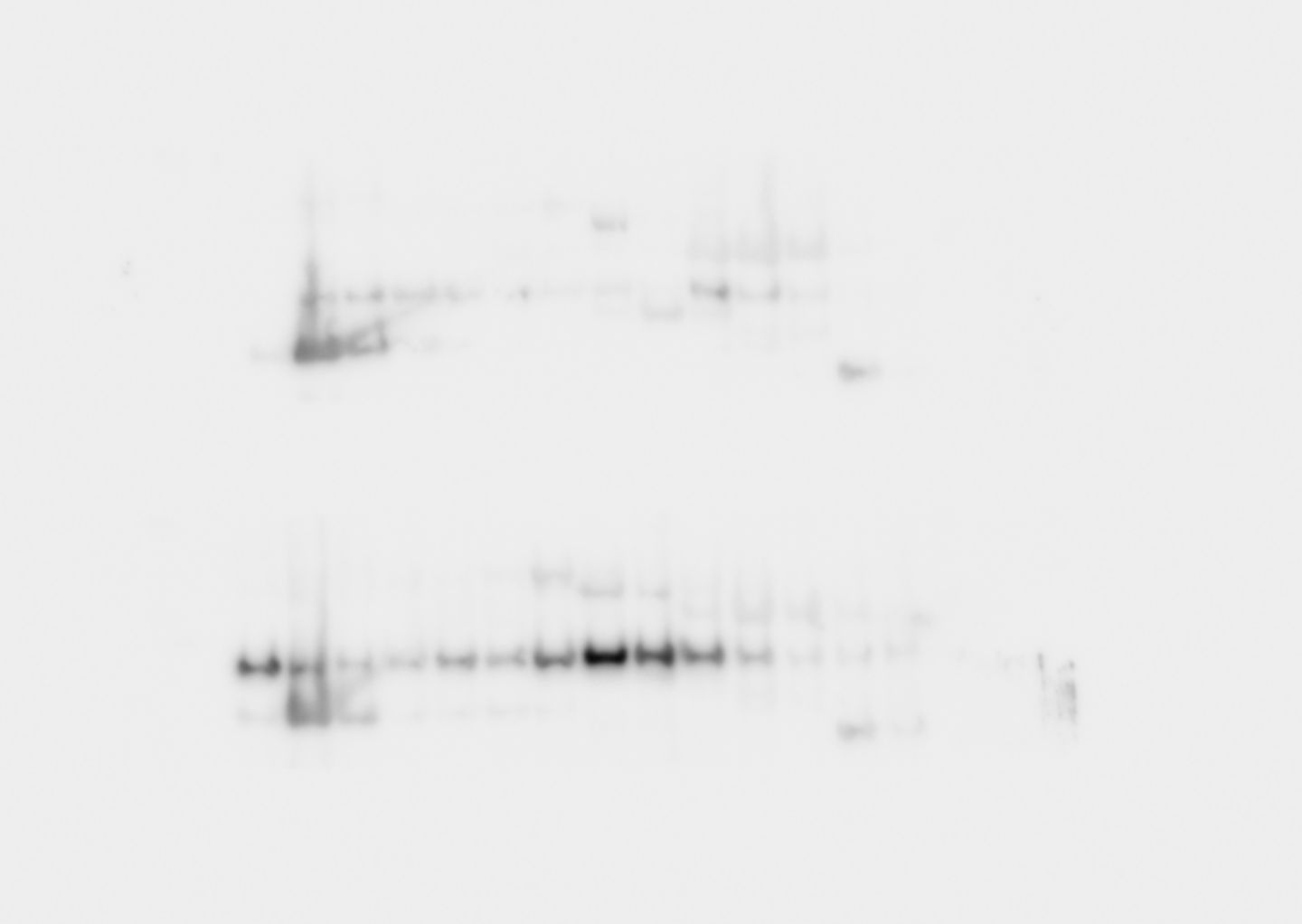

Supplement: Figure 1—source data 2. [file elife-63505-fig1-data2.zip › Figure 1 - Source Data 1/RAW/Western blot.tif]

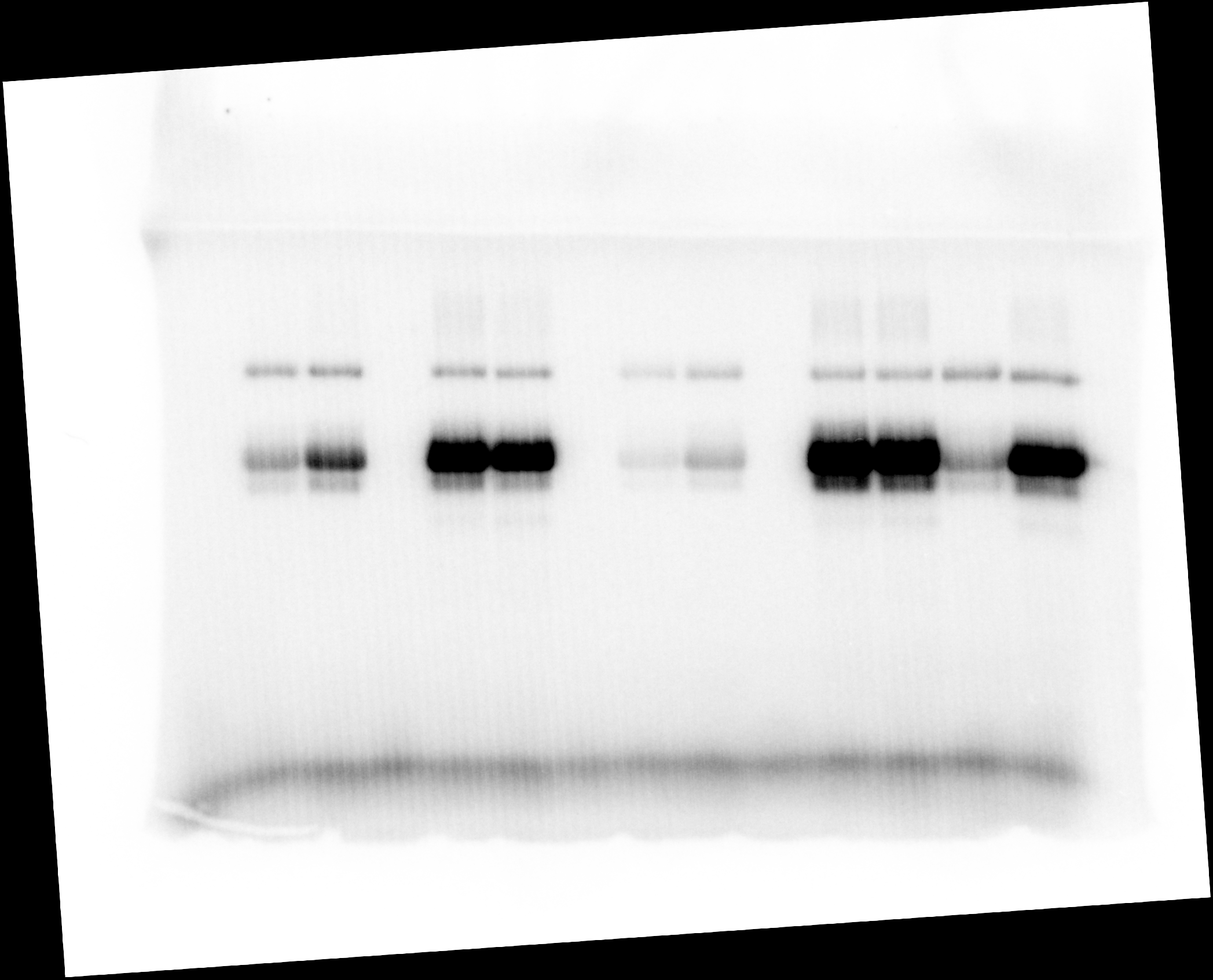

Supplement: Figure 4—source data 2. [file elife-63505-fig4-data2.zip › Figure 4 - Source Data 1/Figure 4b/Adjusted/Contrast_adjusted_Radiometric_replicate1.tif]

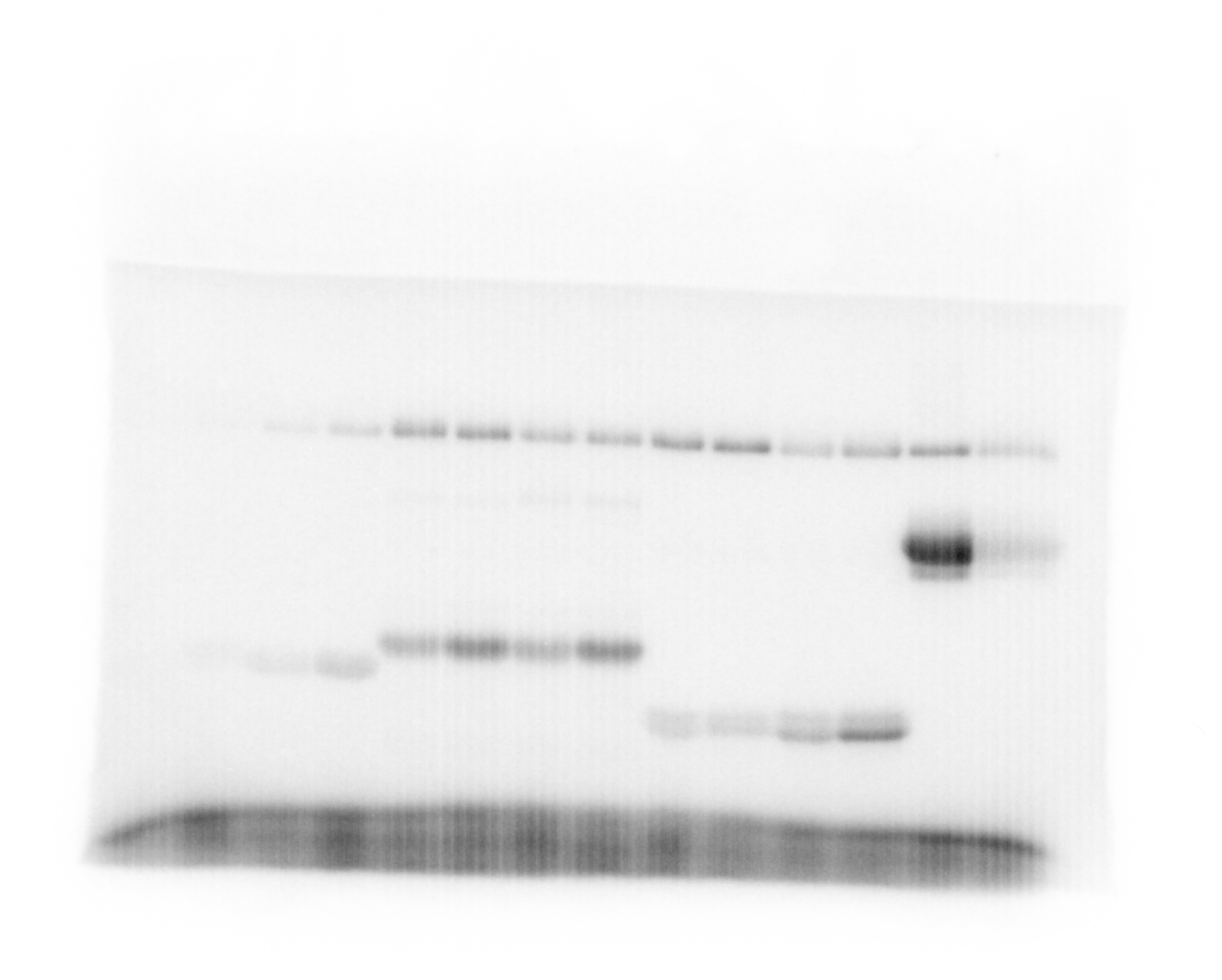

Supplement: Figure 4—source data 2. [file elife-63505-fig4-data2.zip › Figure 4 - Source Data 1/Figure 4b/Adjusted/Contrast_adjusted_Radiometric_replicate2.tif]

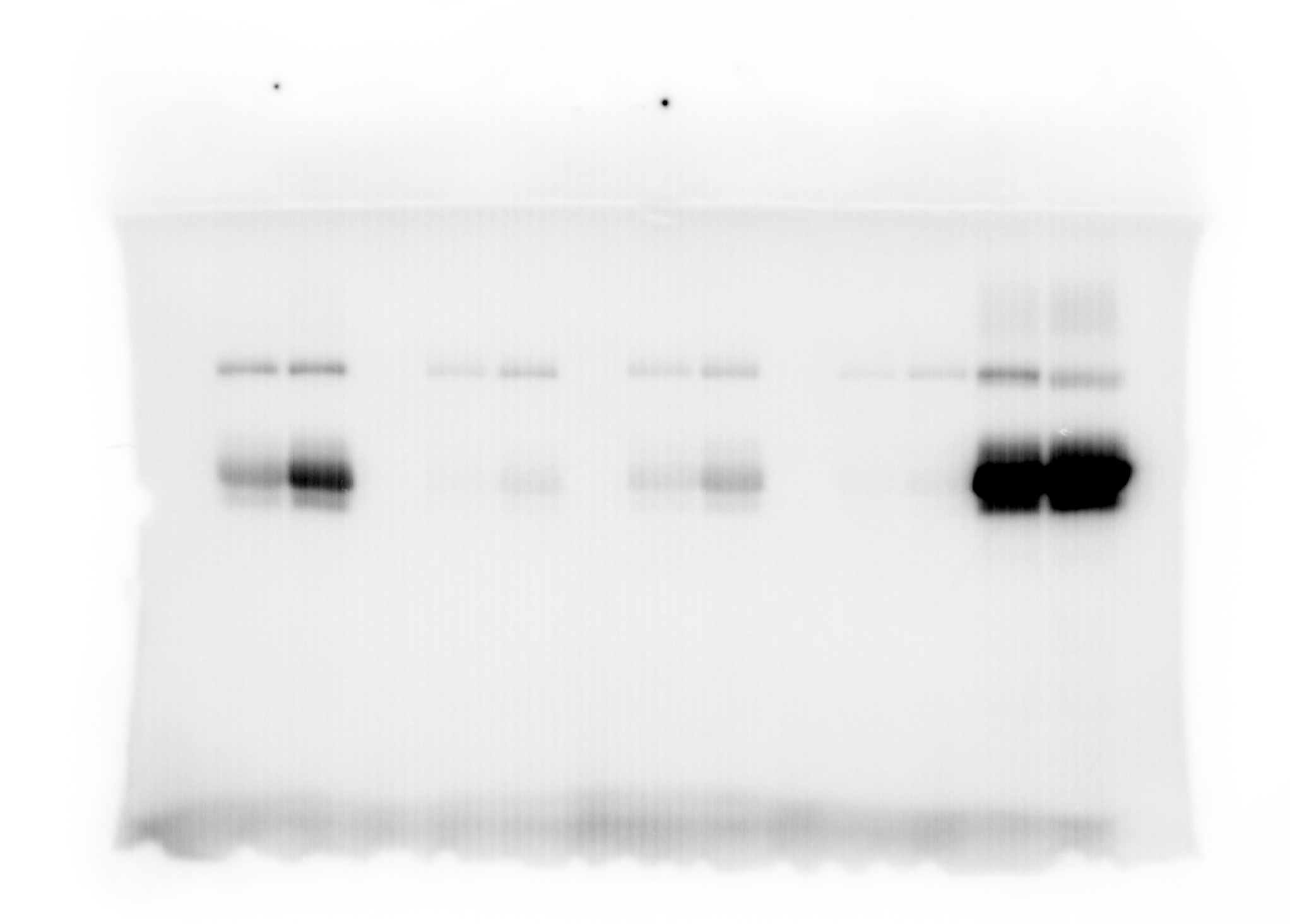

Supplement: Figure 4—source data 2. [file elife-63505-fig4-data2.zip › Figure 4 - Source Data 1/Figure 4b/Adjusted/Contrast_adjusted_Radiometric_replicate3.tif]

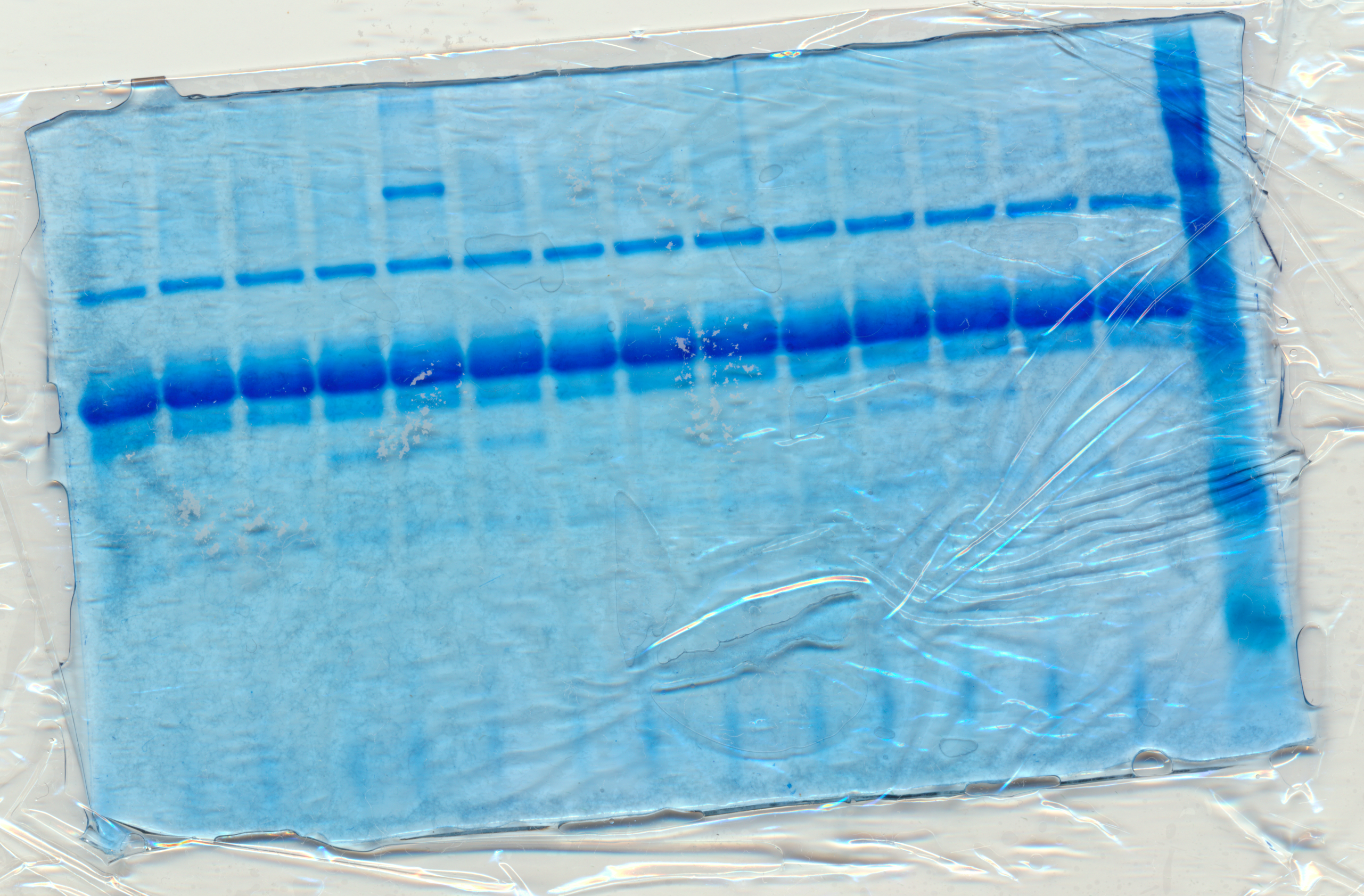

Supplement: Figure 4—source data 2. [file elife-63505-fig4-data2.zip › Figure 4 - Source Data 1/Figure 4b/RAW/Loading_control_replicate 1.tif]

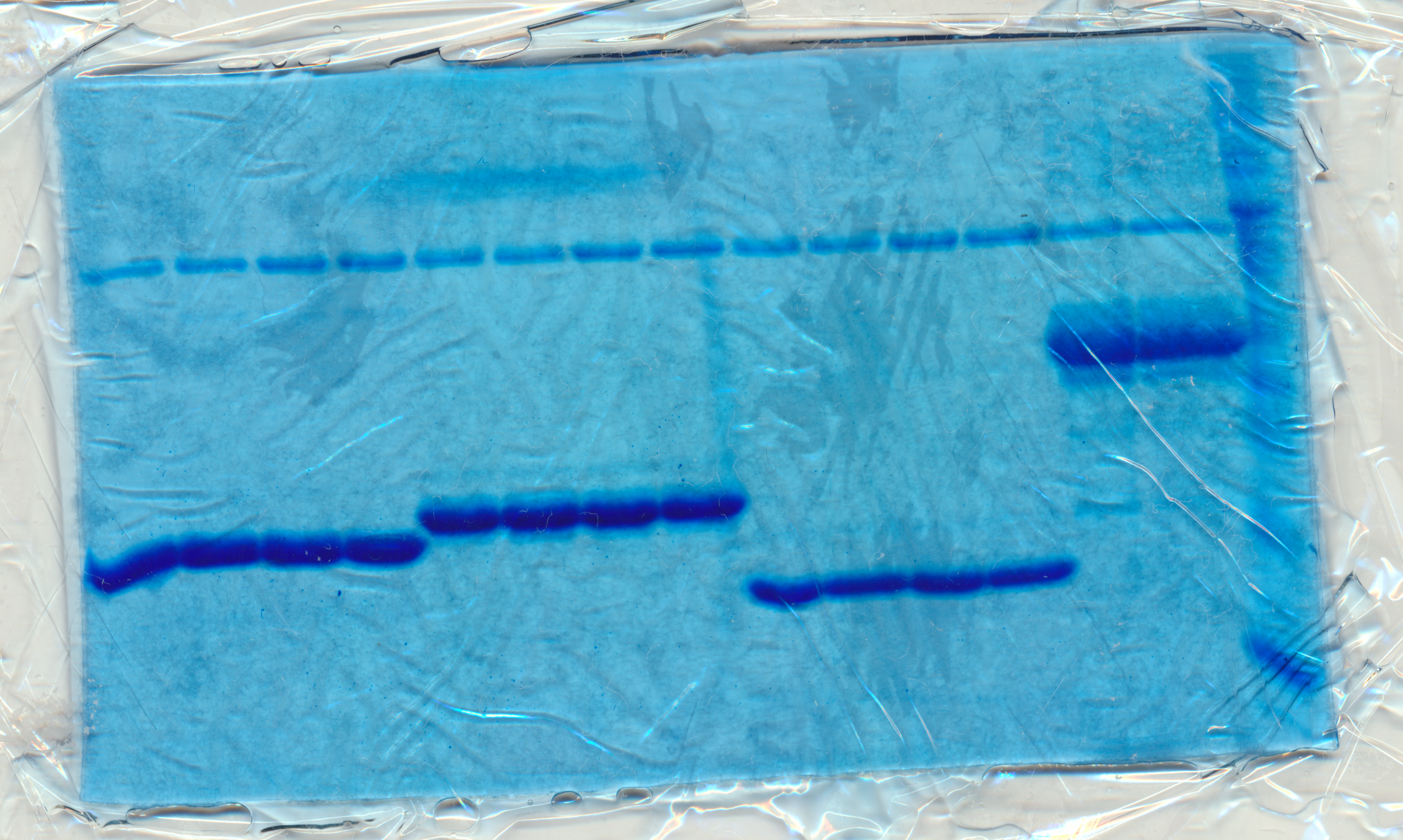

Supplement: Figure 4—source data 2. [file elife-63505-fig4-data2.zip › Figure 4 - Source Data 1/Figure 4b/RAW/Loading_control_replicate 2.tif]

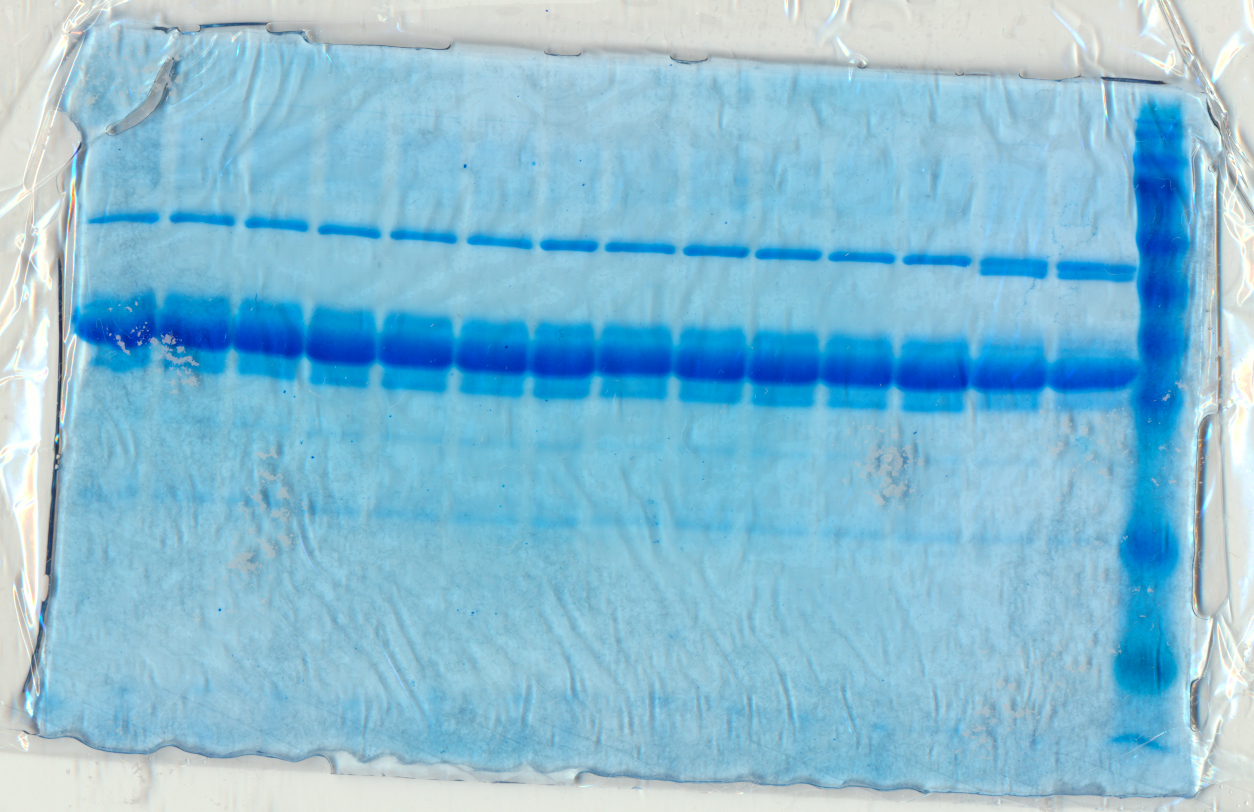

Supplement: Figure 4—source data 2. [file elife-63505-fig4-data2.zip › Figure 4 - Source Data 1/Figure 4b/RAW/Loading_control_replicate 3.tif]

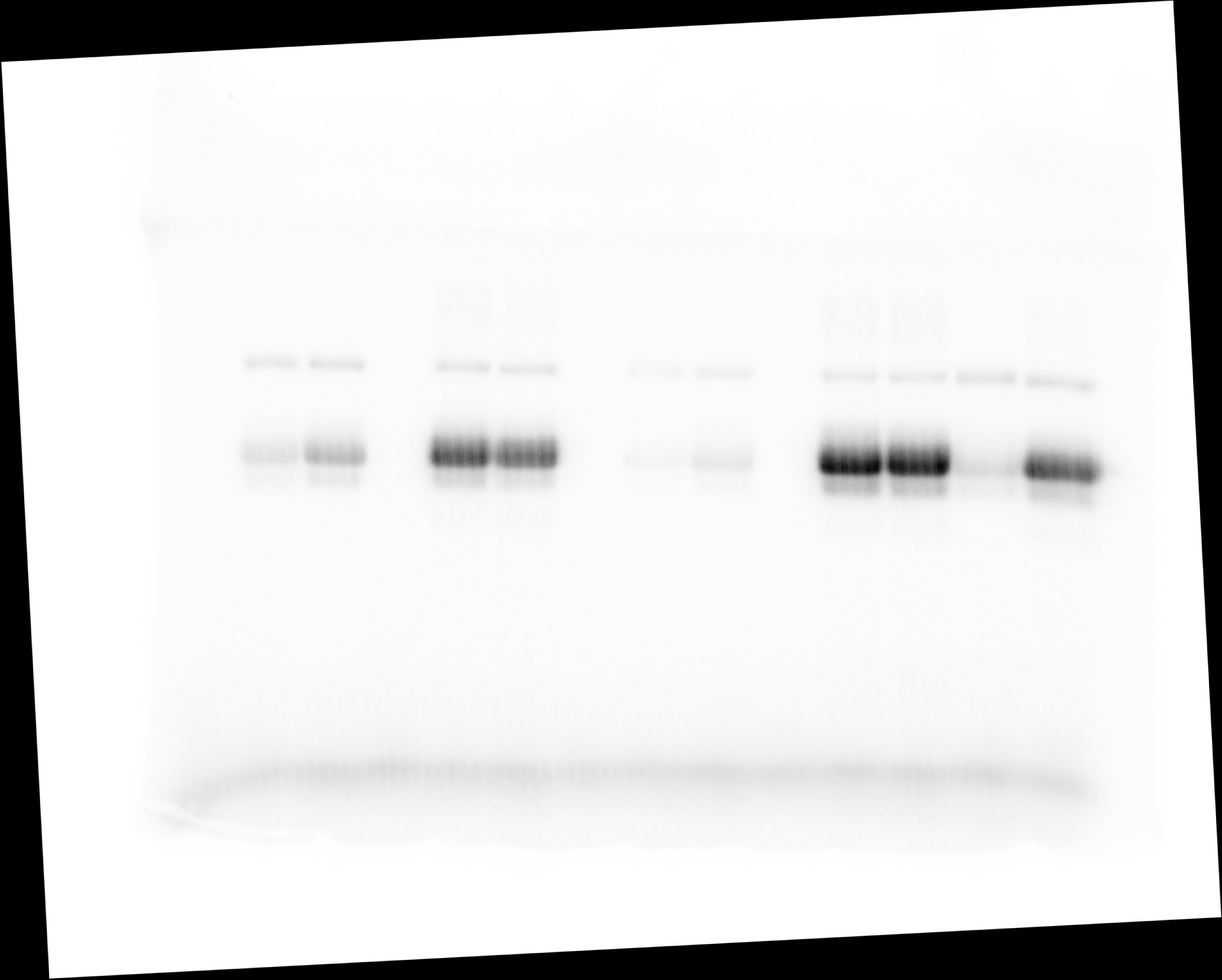

Supplement: Figure 4—source data 2. [file elife-63505-fig4-data2.zip › Figure 4 - Source Data 1/Figure 4b/RAW/Radiometric_replicate1.tif]

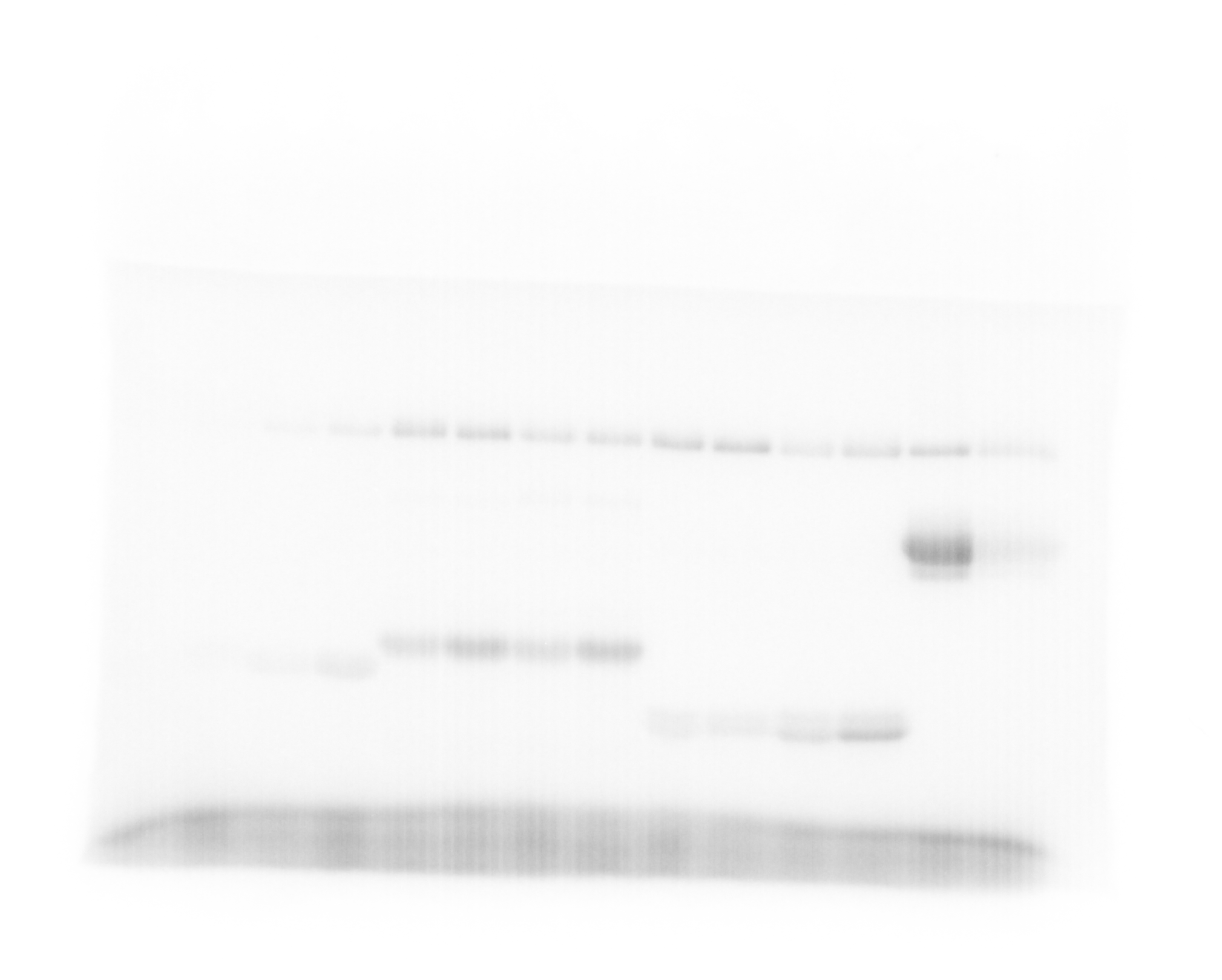

Supplement: Figure 4—source data 2. [file elife-63505-fig4-data2.zip › Figure 4 - Source Data 1/Figure 4b/RAW/Radiometric_replicate2.tif]

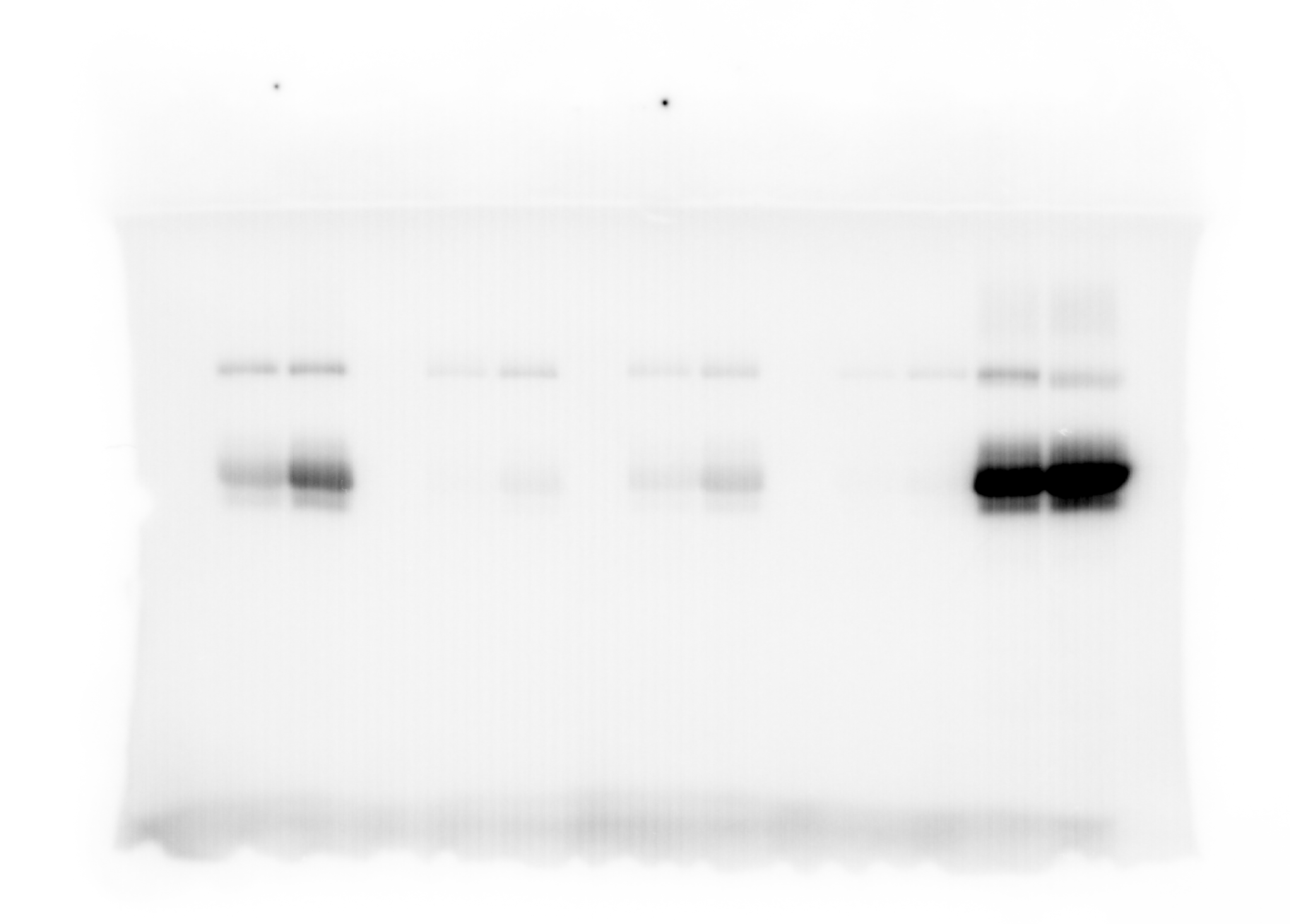

Supplement: Figure 4—source data 2. [file elife-63505-fig4-data2.zip › Figure 4 - Source Data 1/Figure 4b/RAW/Radiometric_replicate3.tif]

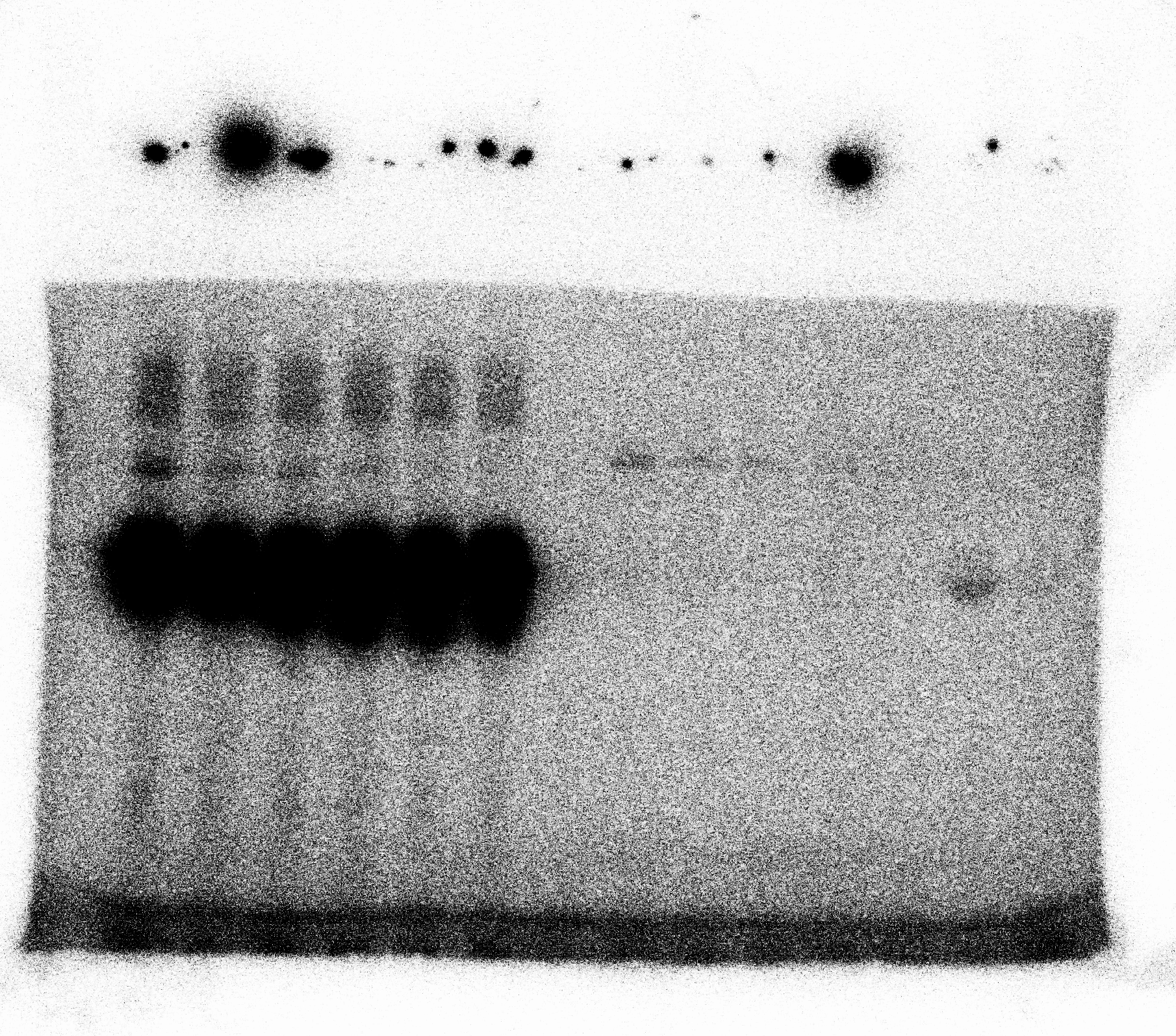

Supplement: Figure 4—source data 2. [file elife-63505-fig4-data2.zip › Figure 4 - Source Data 1/Figure 4c/Adjusted/Contrast_adjusted_Radiometric.tif]

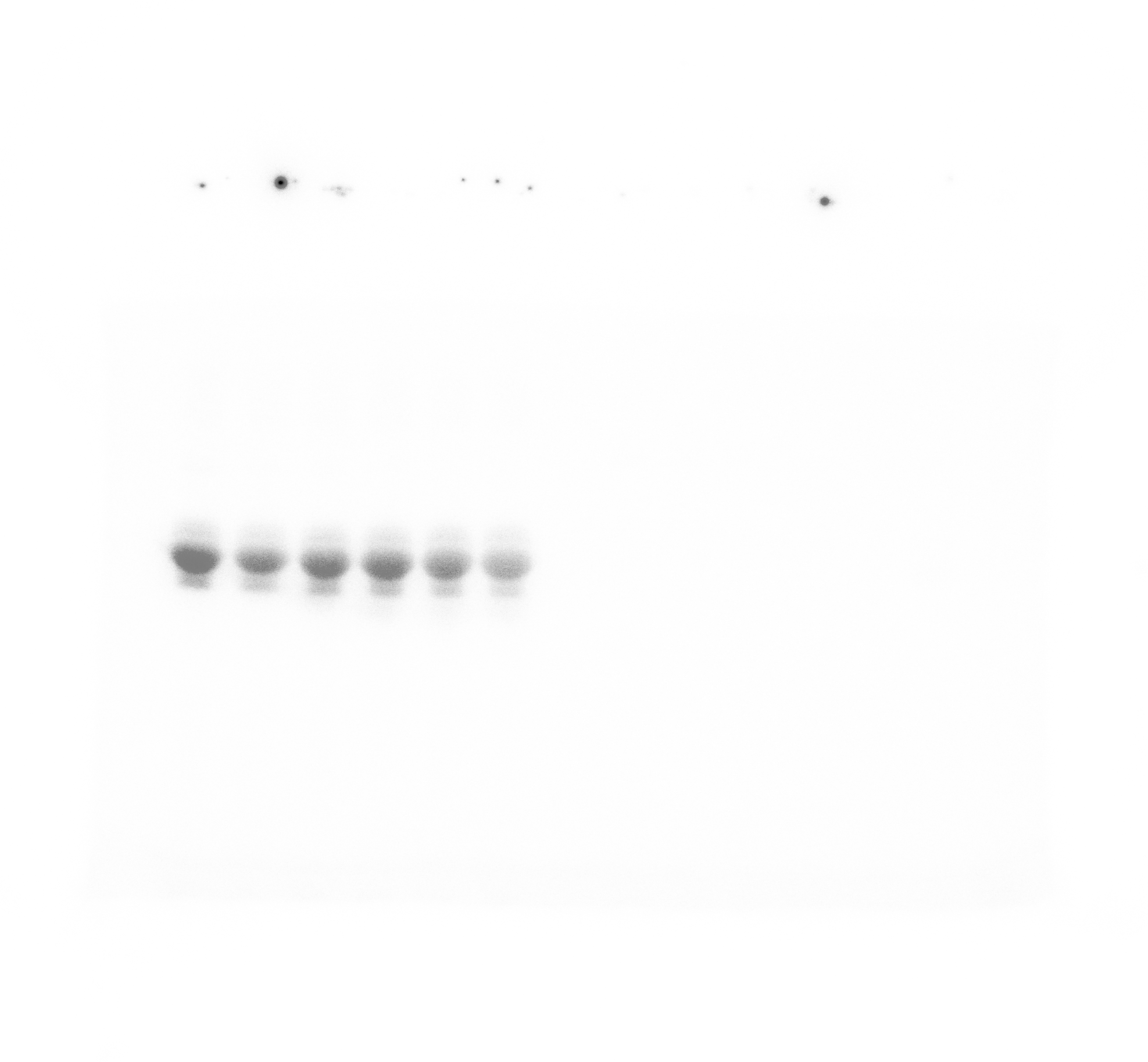

Supplement: Figure 4—source data 2. [file elife-63505-fig4-data2.zip › Figure 4 - Source Data 1/Figure 4c/RAW/Radiometric.tif]

**Figure 5 – figure supplement 1a**


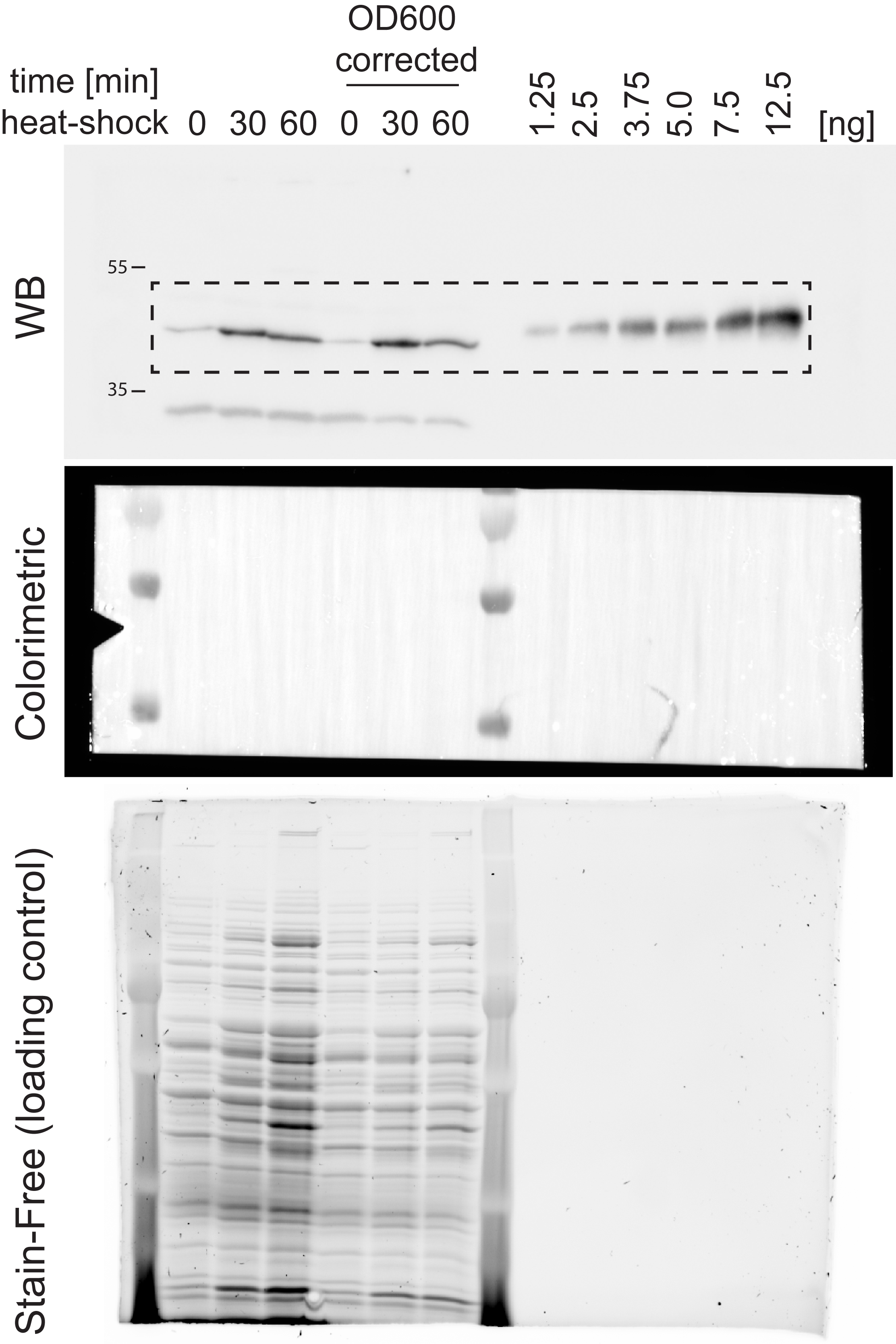

Supplement: Figure 5—figure supplement 1—source data 1. [file elife-63505-fig5-figsupp1-data1.docx]

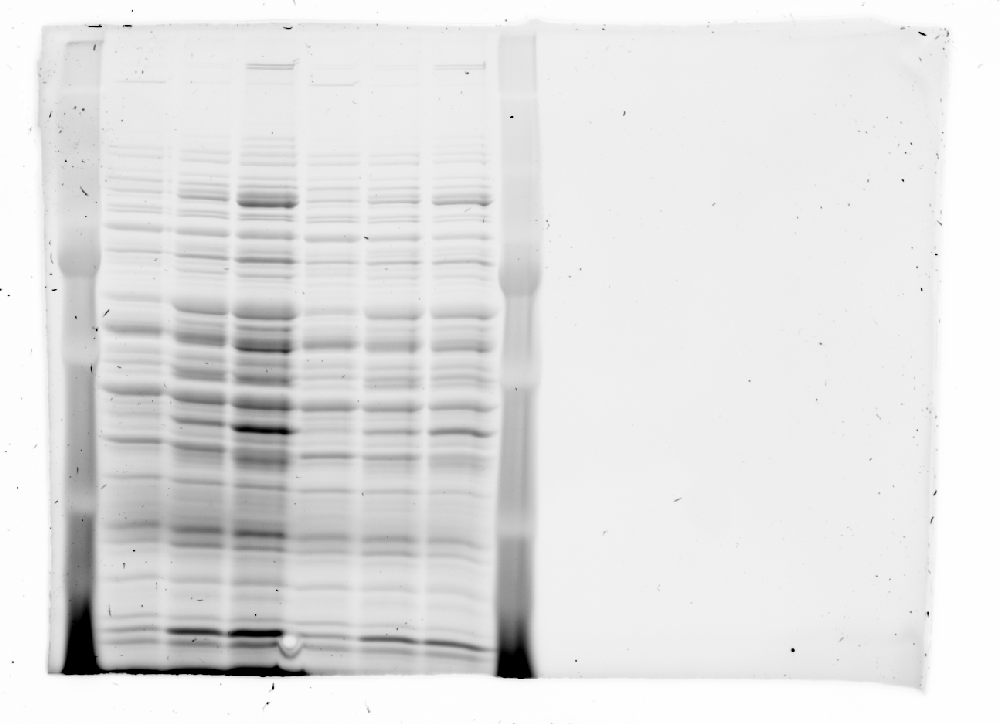

Supplement: Figure 5—figure supplement 1—source data 2. [file elife-63505-fig5-figsupp1-data2.zip › Figure 5 - figure supplement 1 - Source Data 1/Adjusted/Loading control_Stain free_Contrast adjusted.tif]

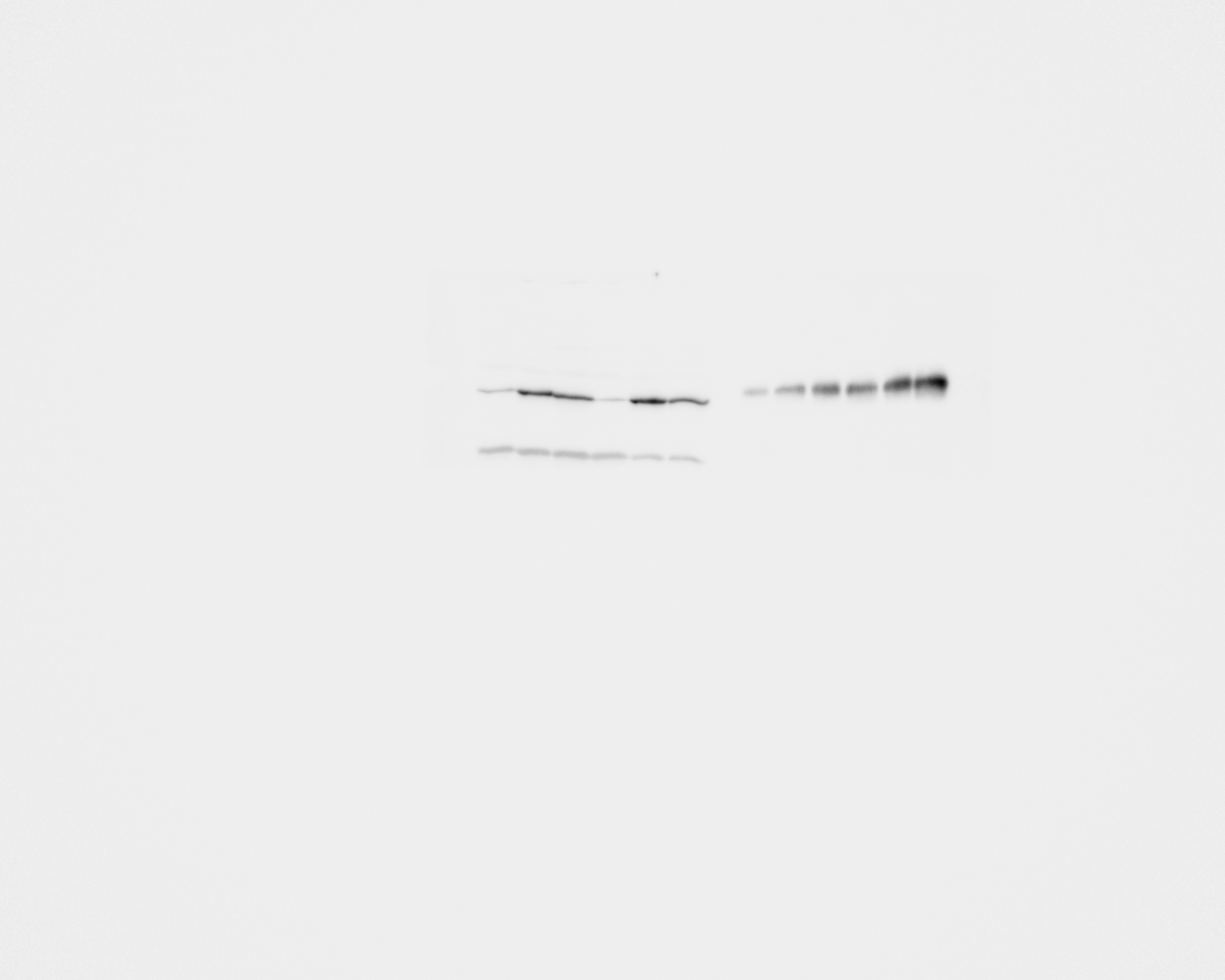

Supplement: Figure 5—figure supplement 1—source data 2. [file elife-63505-fig5-figsupp1-data2.zip › Figure 5 - figure supplement 1 - Source Data 1/Adjusted/Western blot_Contrast adjusted.tif]

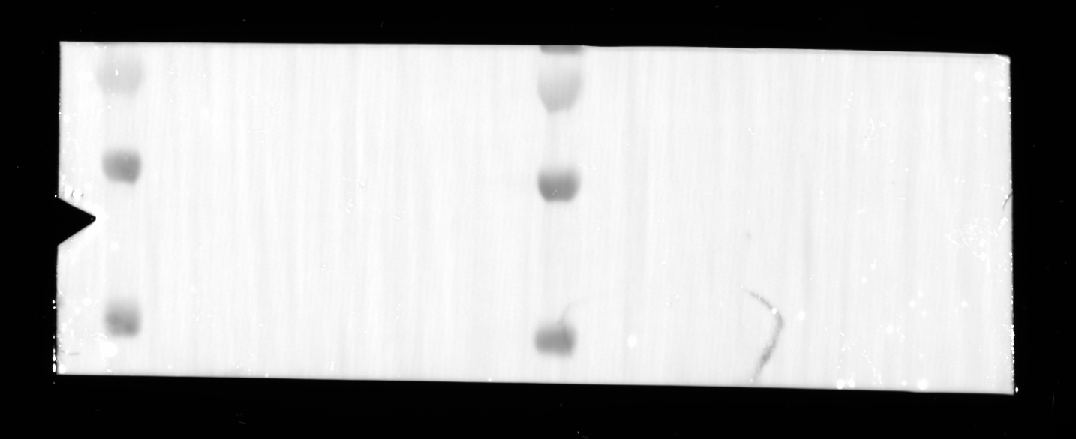

Supplement: Figure 5—figure supplement 1—source data 2. [file elife-63505-fig5-figsupp1-data2.zip › Figure 5 - figure supplement 1 - Source Data 1/RAW/Colorimetric.tif]

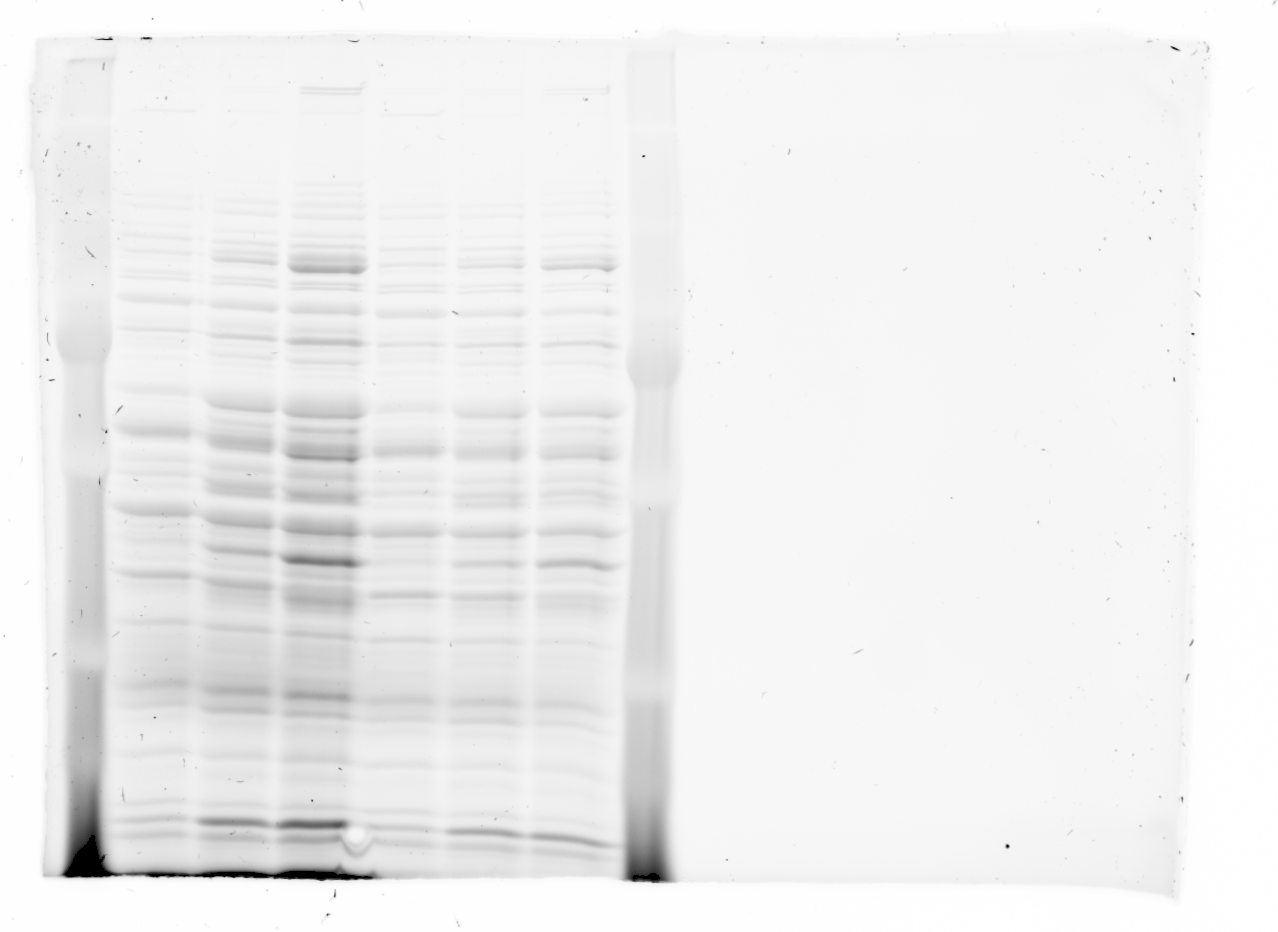

Supplement: Figure 5—figure supplement 1—source data 2. [file elife-63505-fig5-figsupp1-data2.zip › Figure 5 - figure supplement 1 - Source Data 1/RAW/Loading control_Stain free.tif]

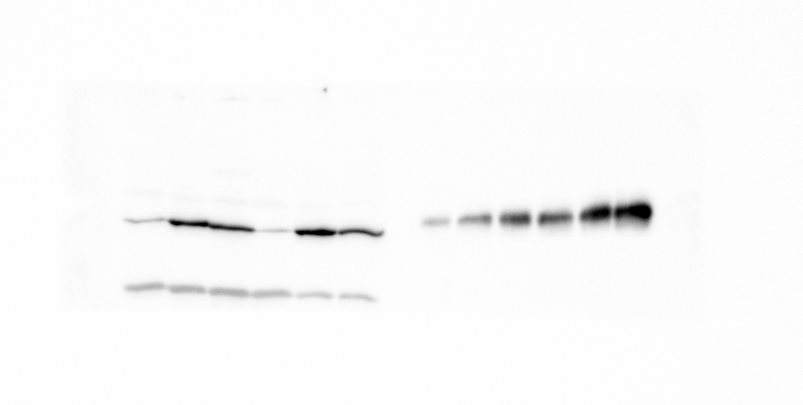

Supplement: Figure 5—figure supplement 1—source data 2. [file elife-63505-fig5-figsupp1-data2.zip › Figure 5 - figure supplement 1 - Source Data 1/RAW/Western blot.tif]

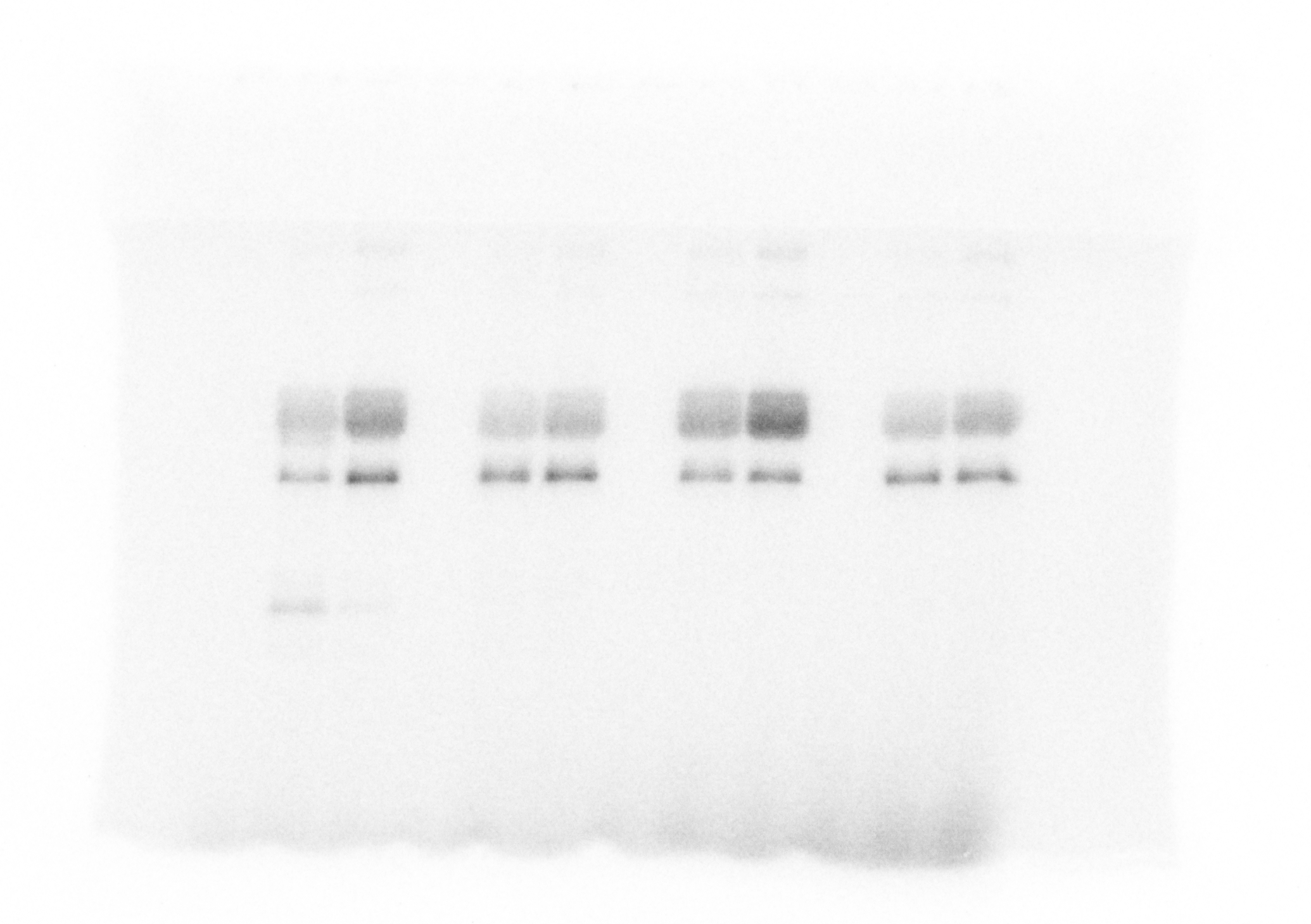

Supplement: Figure 6—figure supplement 1—source data 2. [file elife-63505-fig6-figsupp1-data2.zip › Figure 6 - figure supplement 1 - Source Data 1/Adjusted/Radiometric_Contrast adjusted_Replicate 1.tif]

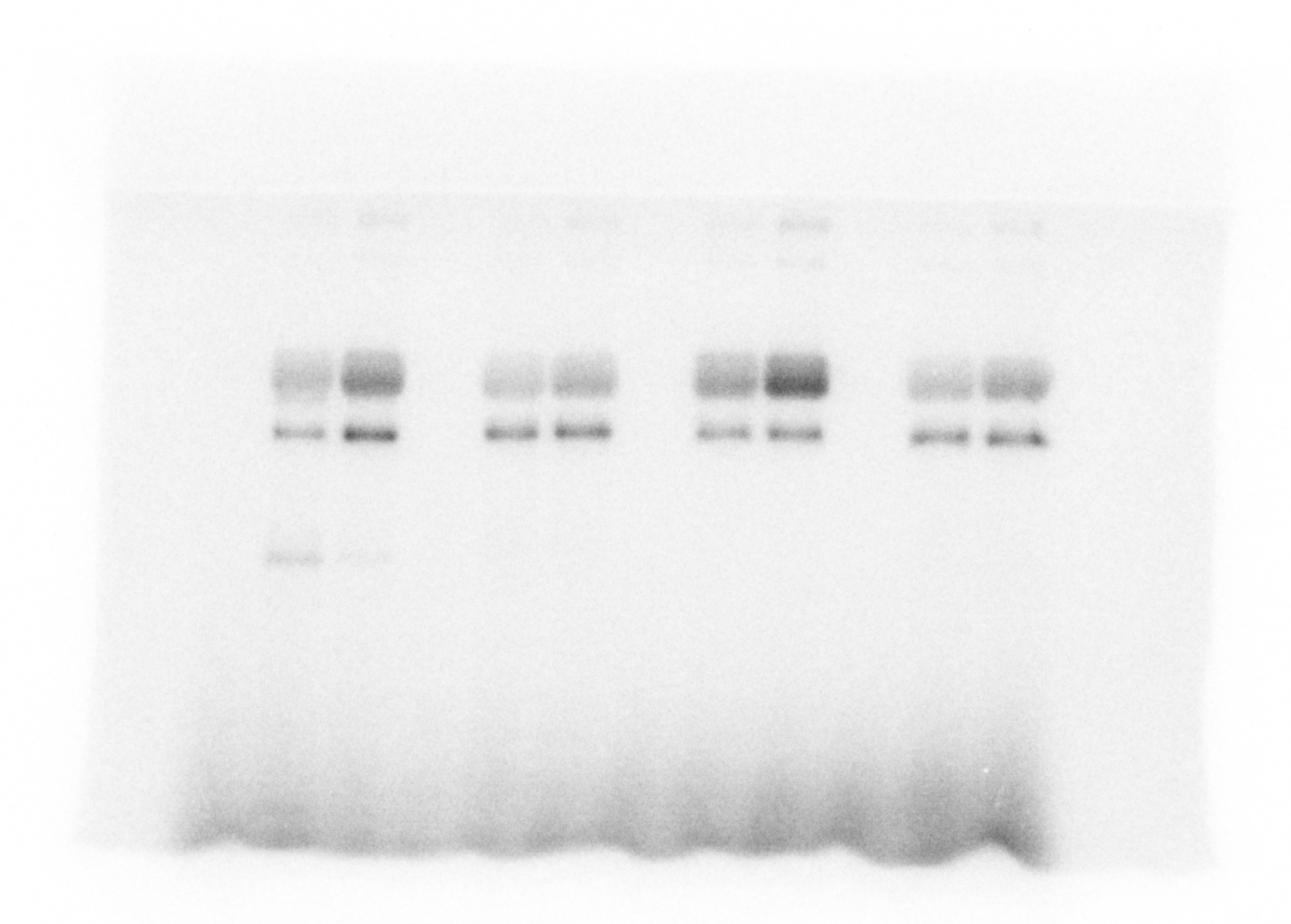

Supplement: Figure 6—figure supplement 1—source data 2. [file elife-63505-fig6-figsupp1-data2.zip › Figure 6 - figure supplement 1 - Source Data 1/Adjusted/Radiometric_Contrast adjusted_Replicate 2.tif]

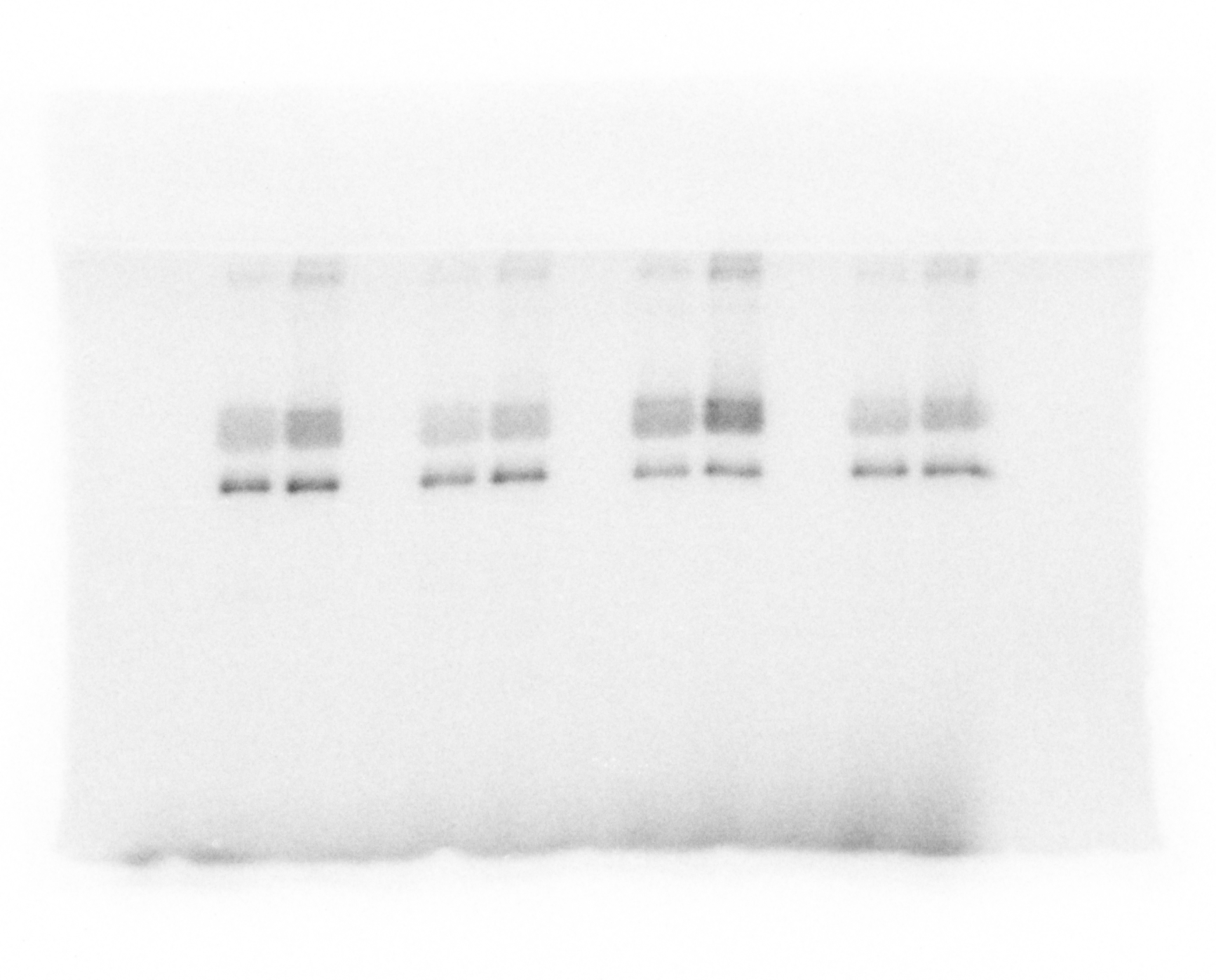

Supplement: Figure 6—figure supplement 1—source data 2. [file elife-63505-fig6-figsupp1-data2.zip › Figure 6 - figure supplement 1 - Source Data 1/Adjusted/Radiometric_Contrast adjusted_Replicate 3.tif]

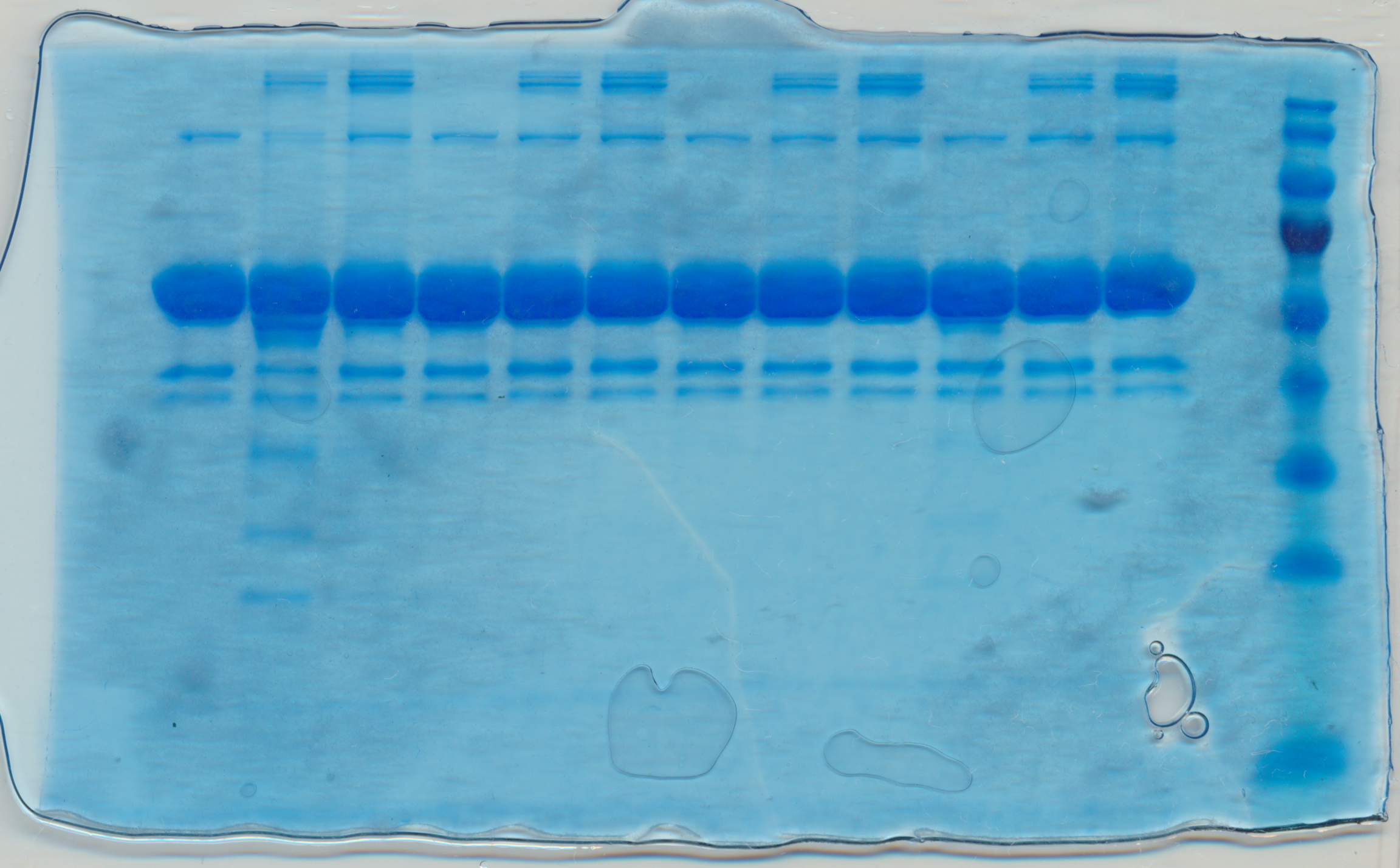

Supplement: Figure 6—figure supplement 1—source data 2. [file elife-63505-fig6-figsupp1-data2.zip › Figure 6 - figure supplement 1 - Source Data 1/RAW/Coomassie_Replicate 1.tif]

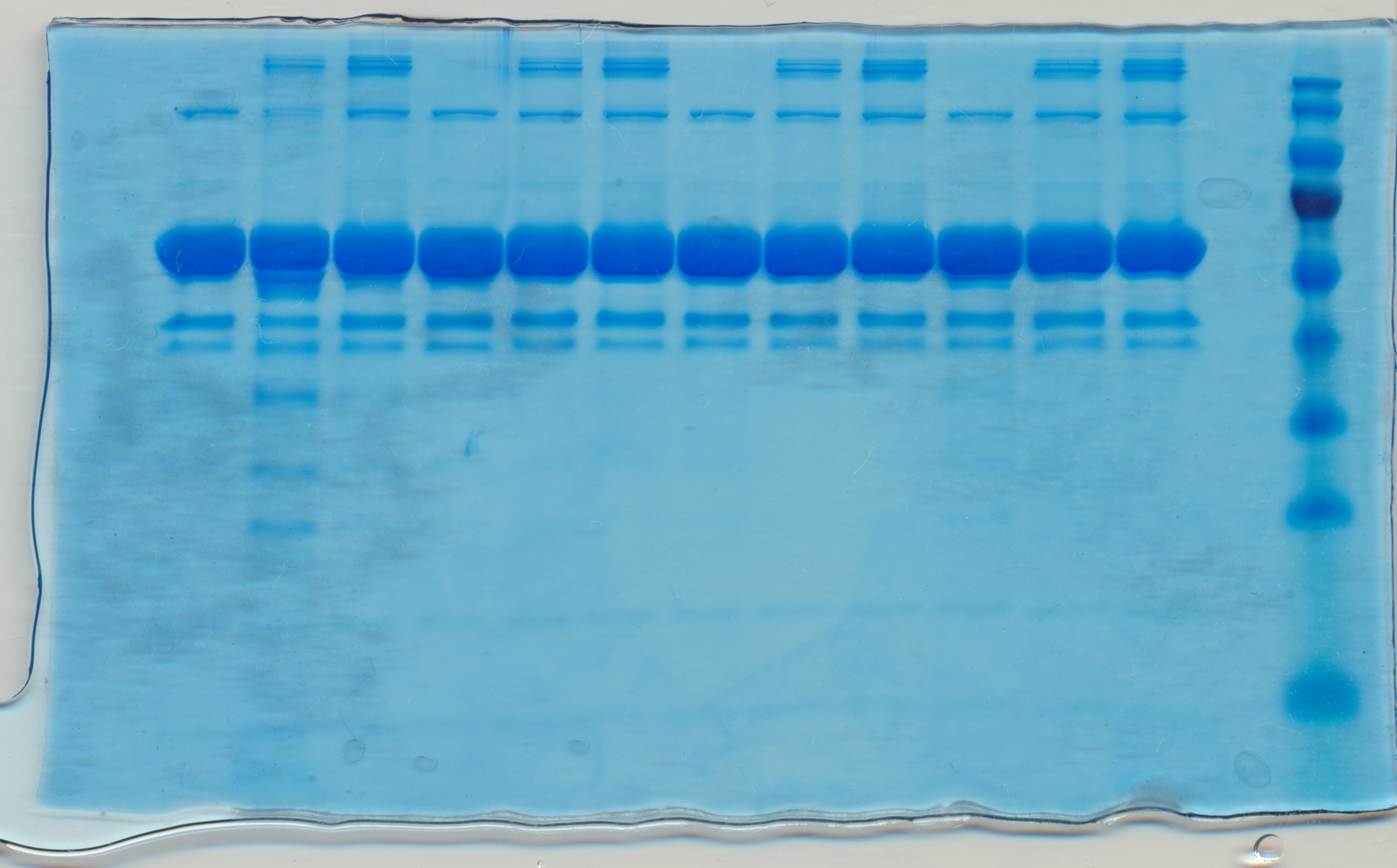

Supplement: Figure 6—figure supplement 1—source data 2. [file elife-63505-fig6-figsupp1-data2.zip › Figure 6 - figure supplement 1 - Source Data 1/RAW/Coomassie_Replicate 2.tif]

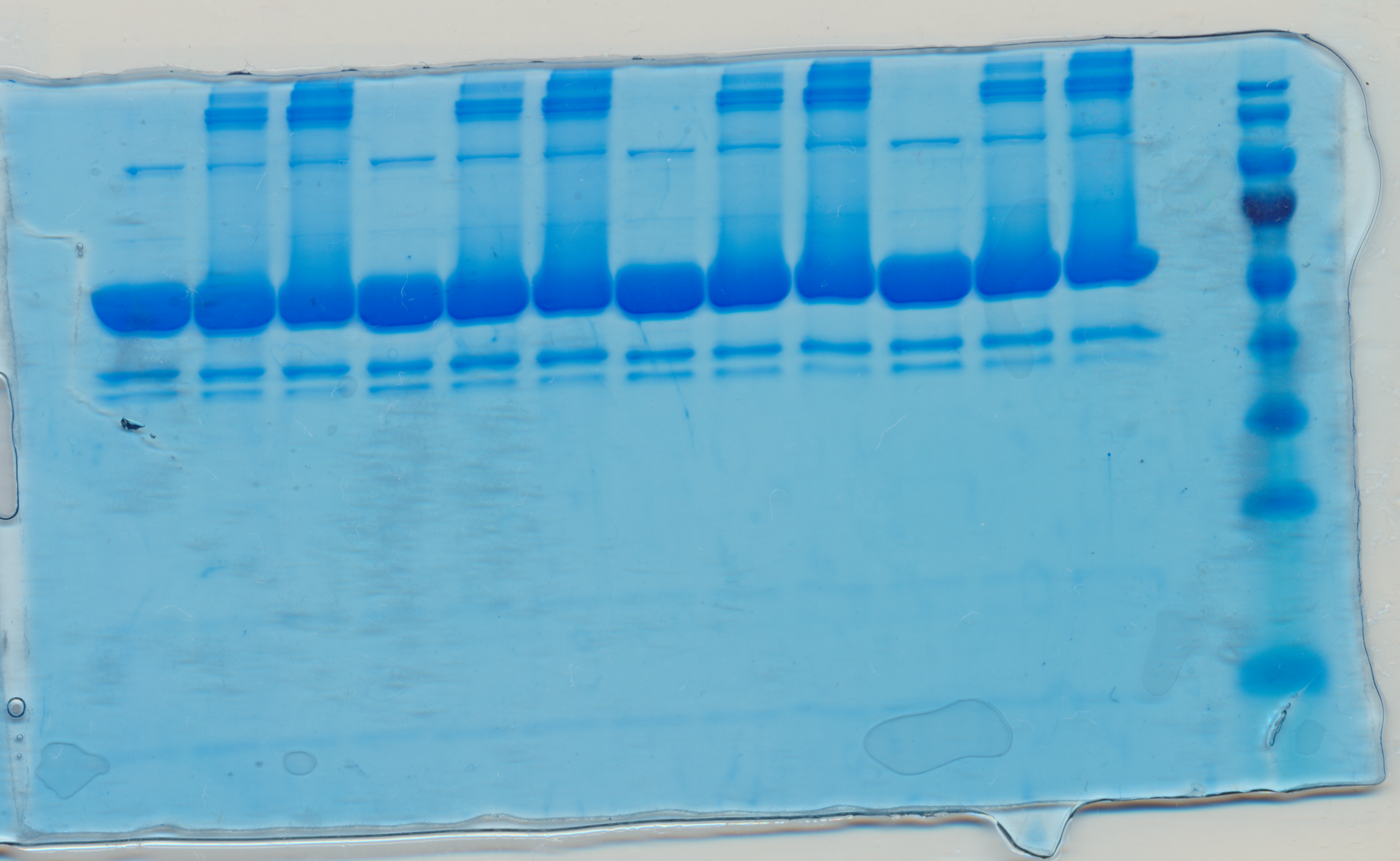

Supplement: Figure 6—figure supplement 1—source data 2. [file elife-63505-fig6-figsupp1-data2.zip › Figure 6 - figure supplement 1 - Source Data 1/RAW/Coomassie_Replicate 3.tif]

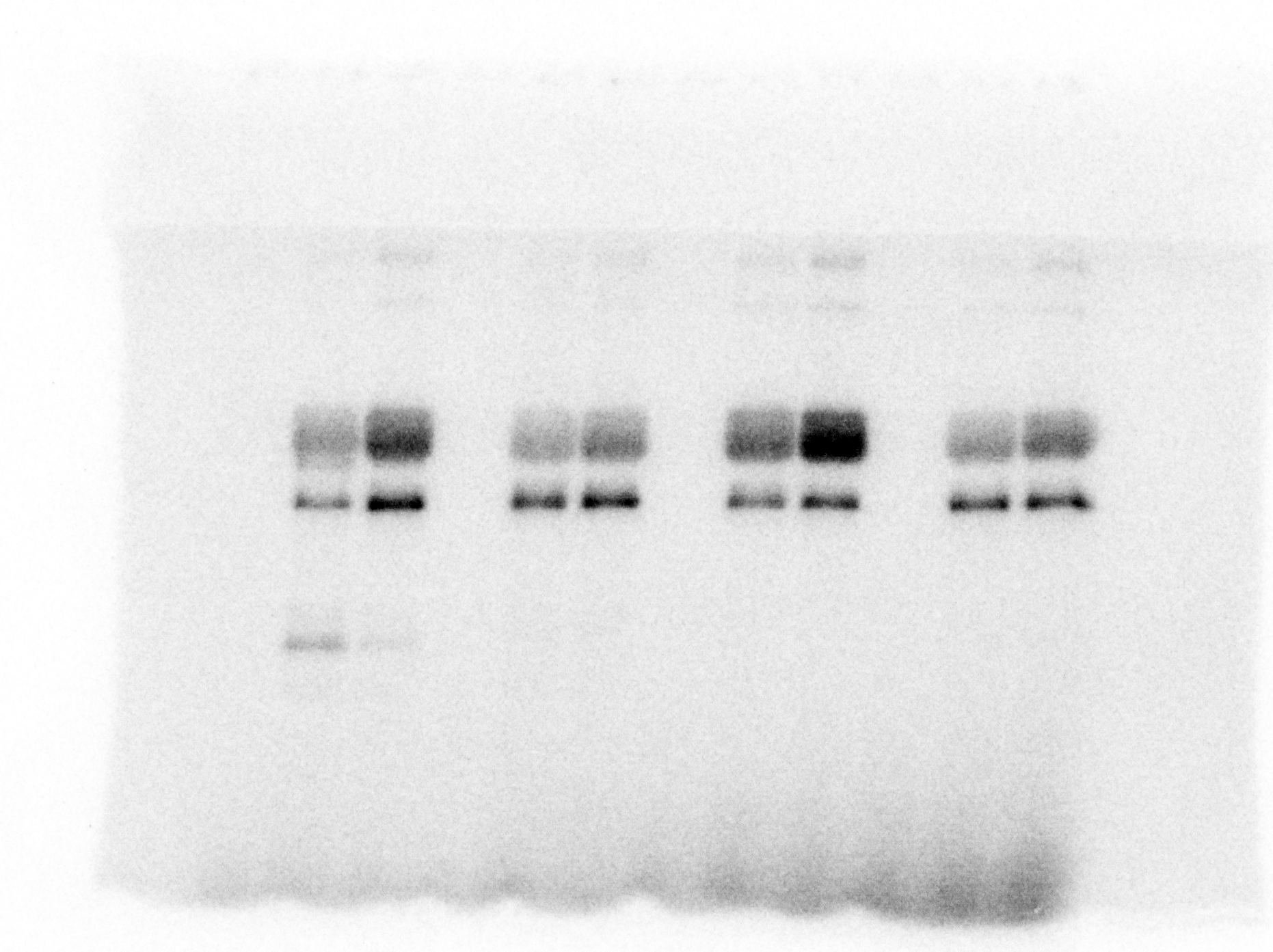

Supplement: Figure 6—figure supplement 1—source data 2. [file elife-63505-fig6-figsupp1-data2.zip › Figure 6 - figure supplement 1 - Source Data 1/RAW/Radiometric_Replicate 1.tif]

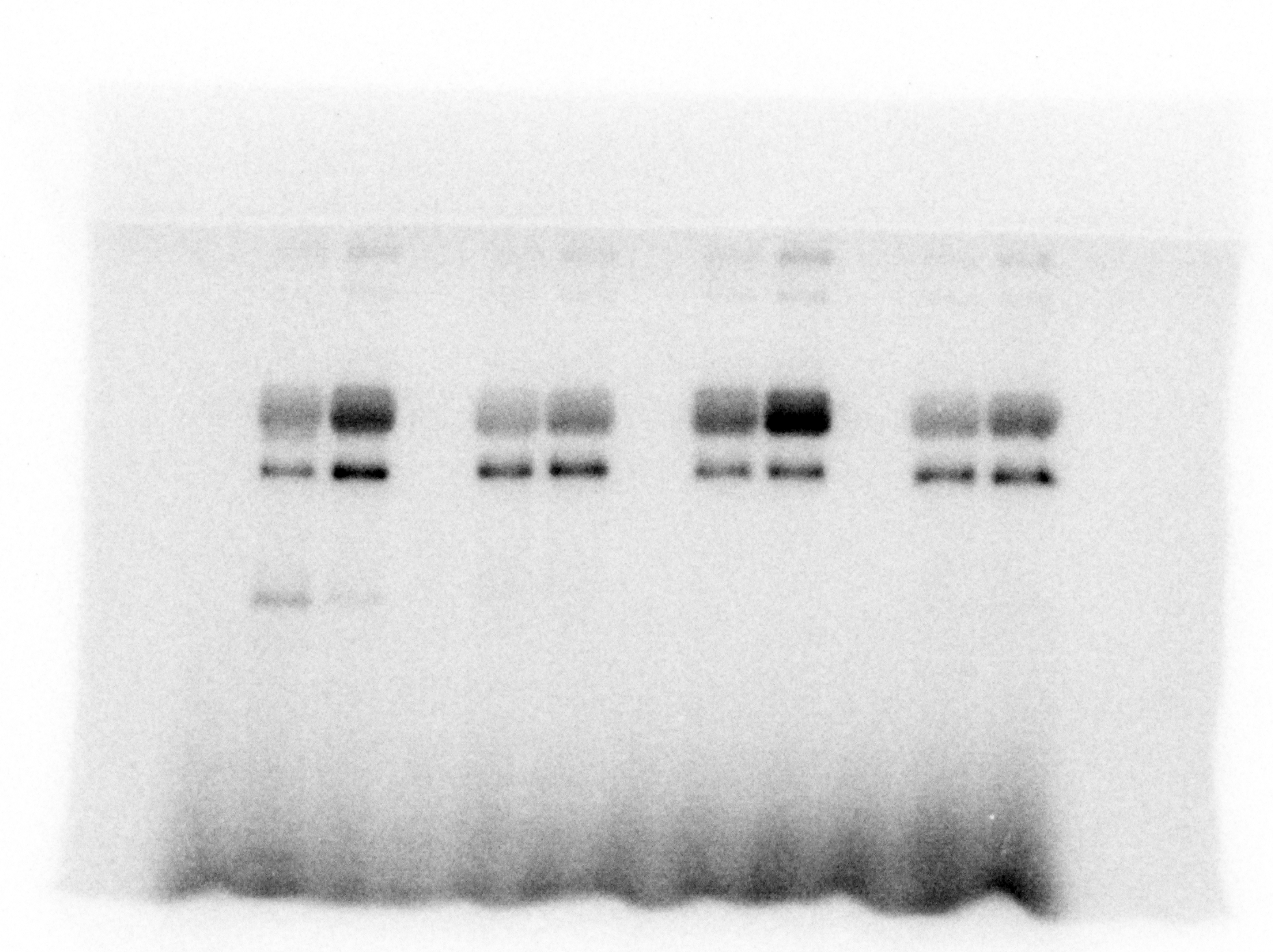

Supplement: Figure 6—figure supplement 1—source data 2. [file elife-63505-fig6-figsupp1-data2.zip › Figure 6 - figure supplement 1 - Source Data 1/RAW/Radiometric_Replicate 2.tif]

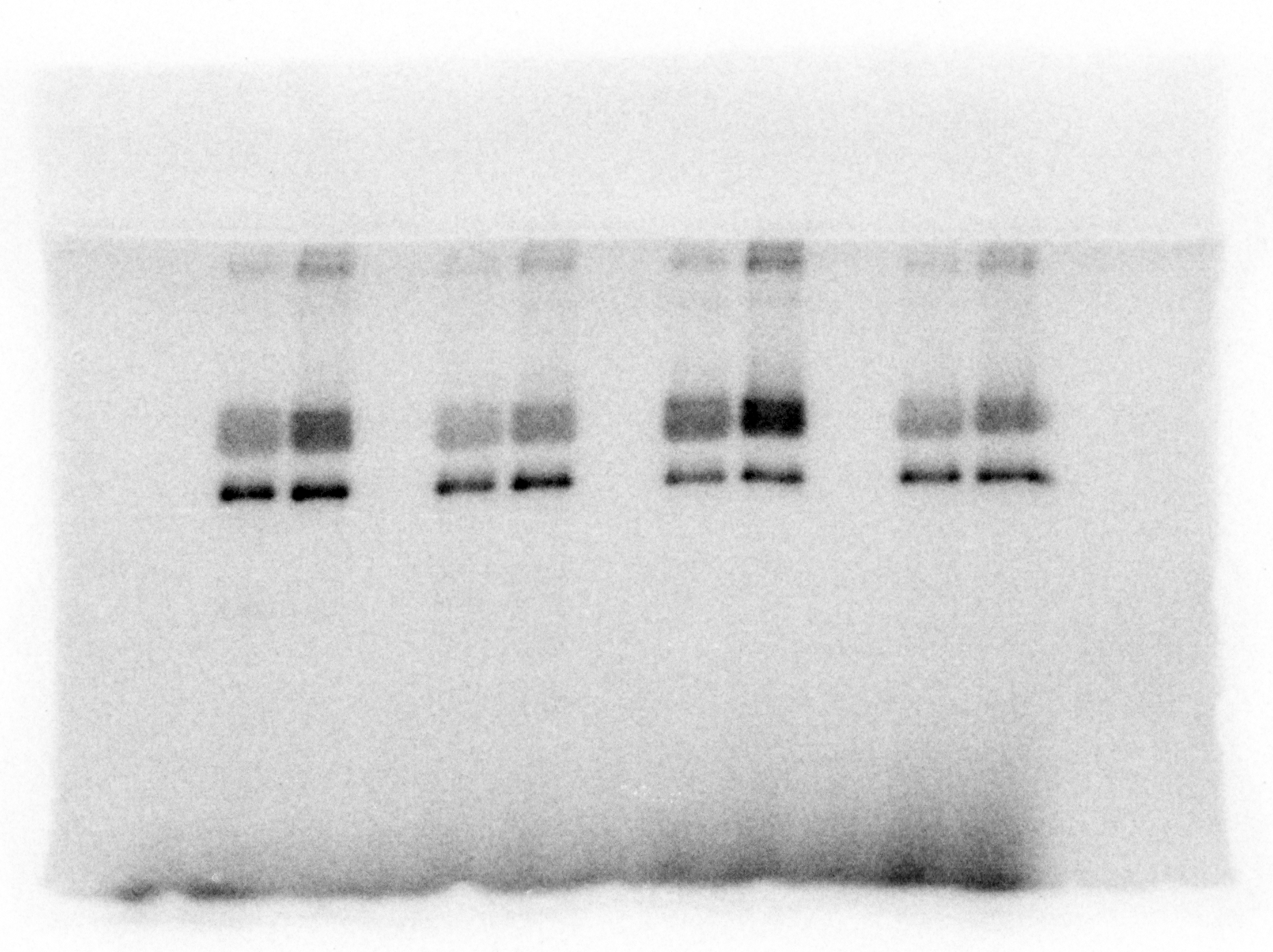

Supplement: Figure 6—figure supplement 1—source data 2. [file elife-63505-fig6-figsupp1-data2.zip › Figure 6 - figure supplement 1 - Source Data 1/RAW/Radiometric_Replicate 3.tif]

**Figure 6 – figure supplement 2b**


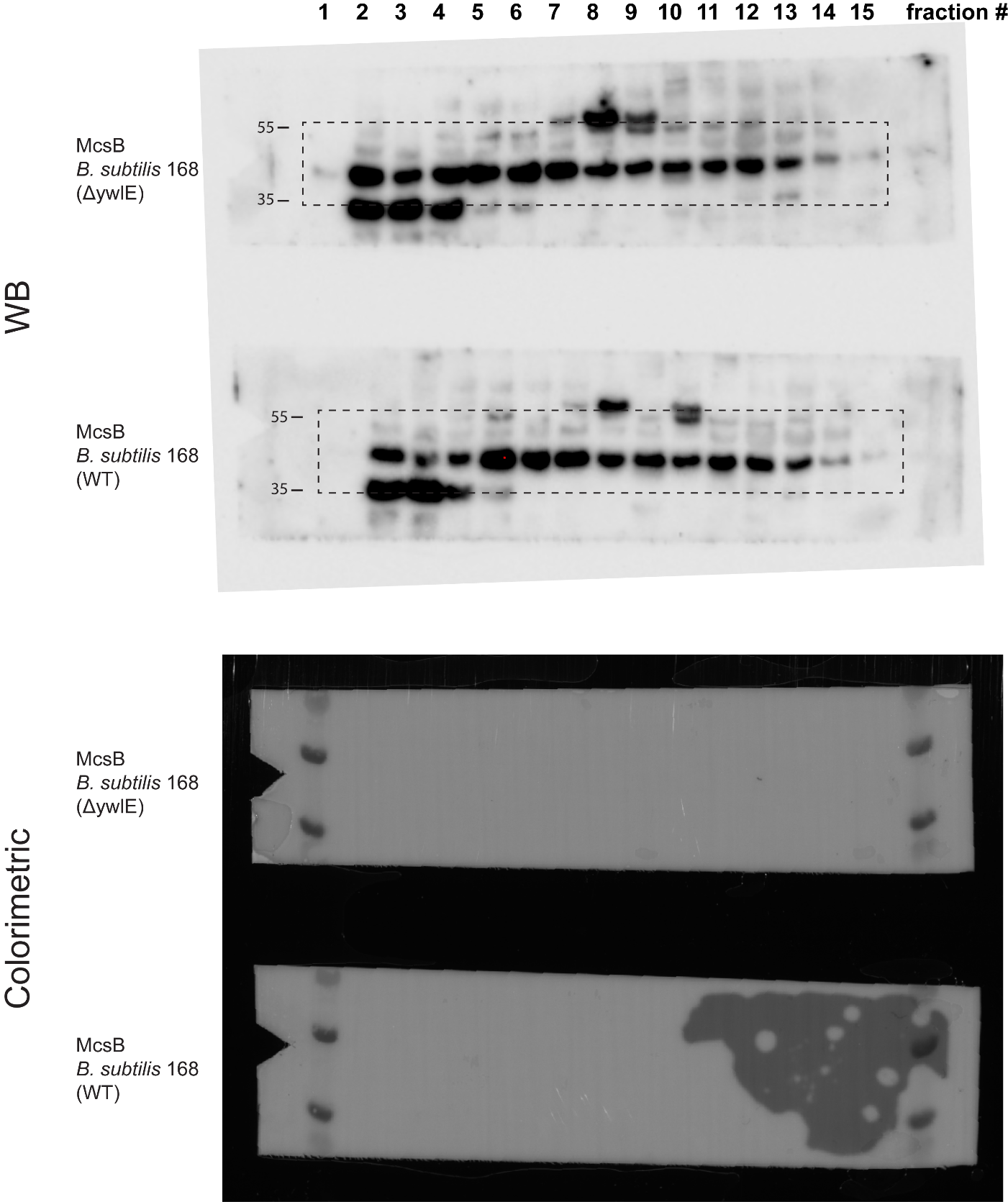

Supplement: Figure 6—figure supplement 2—source data 2. [file elife-63505-fig6-figsupp2-data2.docx]

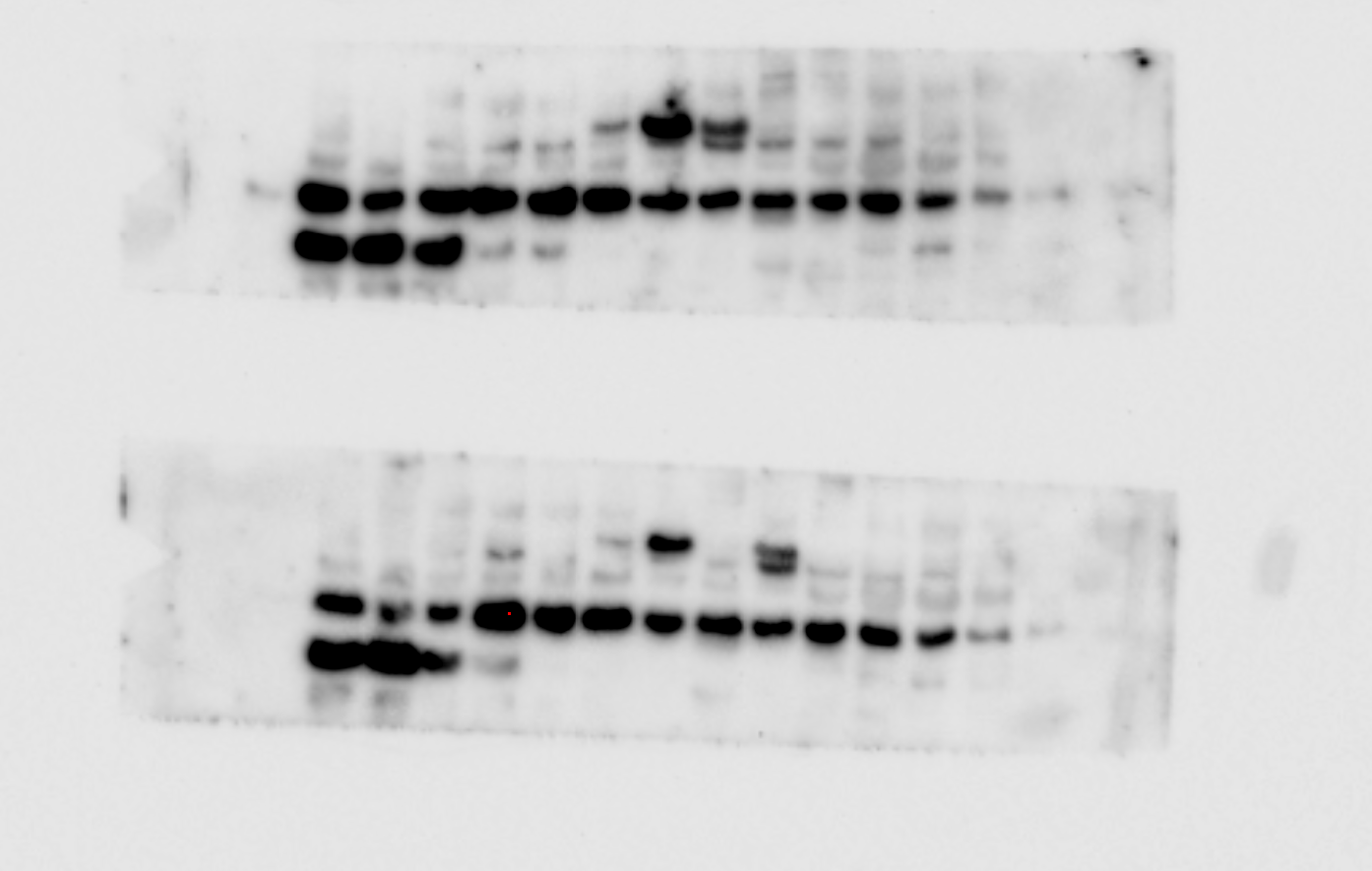

Supplement: Figure 6—figure supplement 2—source data 3. [file elife-63505-fig6-figsupp2-data3.zip › Figure 6 - figure supplement 2 - Source Data 2/Adjusted/Western Blot_Contrast adjusted.tif]

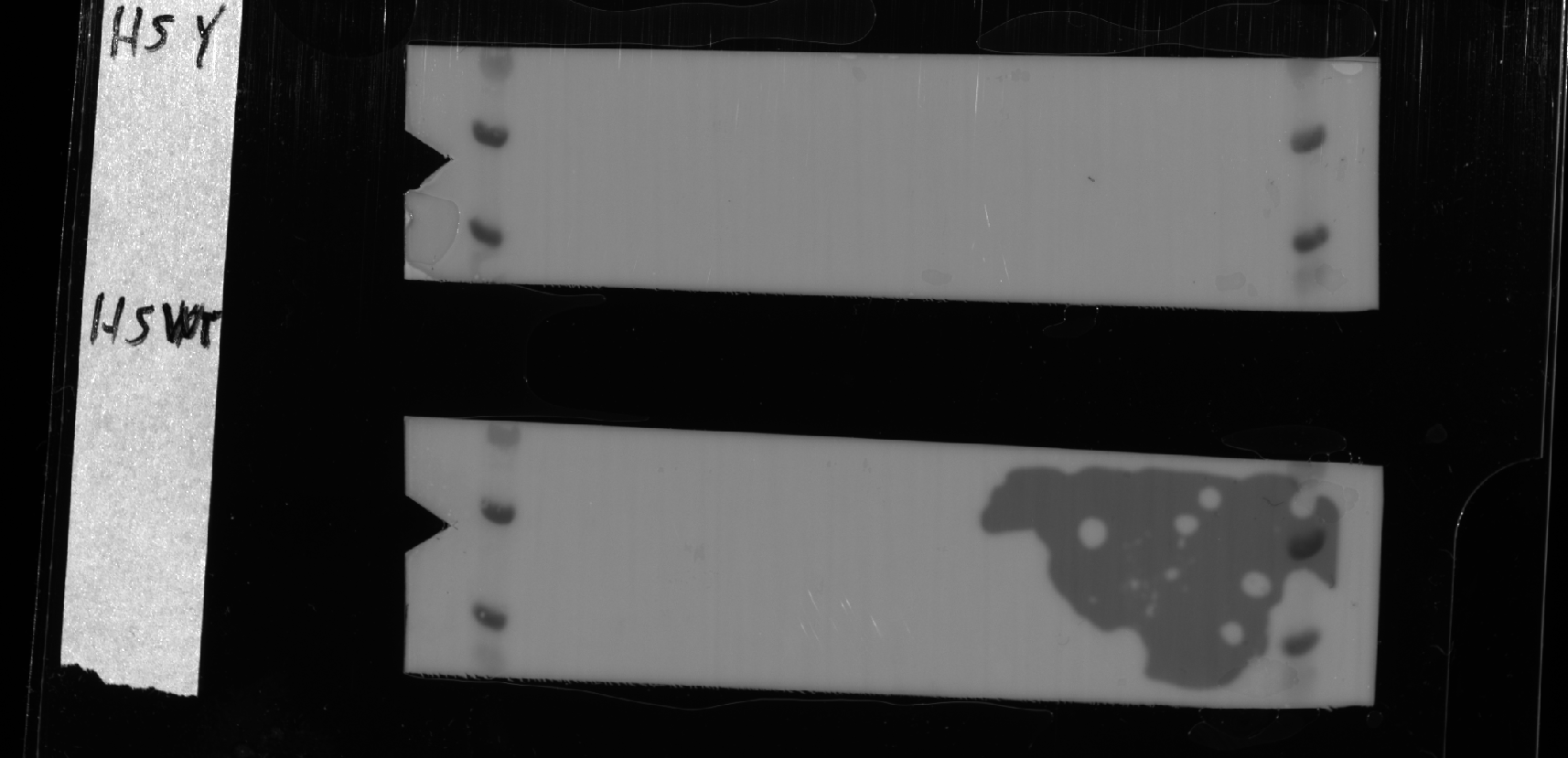

Supplement: Figure 6—figure supplement 2—source data 3. [file elife-63505-fig6-figsupp2-data3.zip › Figure 6 - figure supplement 2 - Source Data 2/RAW/Colorimetric.tif]

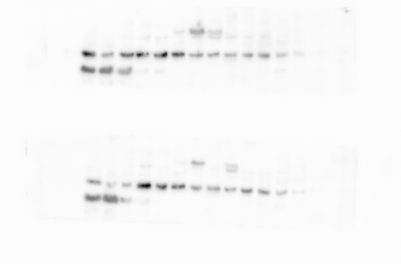

Supplement: Figure 6—figure supplement 2—source data 3. [file elife-63505-fig6-figsupp2-data3.zip › Figure 6 - figure supplement 2 - Source Data 2/RAW/Western Blot.tif]
