# Supplementary material for: McsB forms a gated kinase chamber to mark aberrant bacterial proteins for degradation
Source: eLife. 2021 Jul 30;10:e63505. doi: 10.7554/eLife.63505 (PMC8370763; doi:10.7554/eLife.63505)
Supplement: Figure 3—source data 1. [file elife-63505-fig3-data1.docx]

**Mass photometry – Statistics summary**

**Figure 3 a**

10 nM: N = 371 molecules from 5 technical replicates

50 nM: N = 1766 molecules from 5 technical replicates

100 nM: N = 4445 molecules from 5 technical replicates

500 nM: N = 12005 molecules from 5 technical replicates

**Figure 3 b**

10 nM: N = 1891 molecules from 10 technical replicates

50 nM: N = 5049 molecules from 10 technical replicates

100 nM: N = 10494 molecules from 9 technical replicates

**Figure 3 d**

McsB alone: 16490 molecules from 8 technical replicates

McsB + YwlE: 18938 molecules from 9 technical replicates

**Figure 3 f**

300 nM McsB: 7116 molecules from 6 technical replicates

+ 0.01 mM pArg: 7583 molecules from 6 technical replicates

+ 0.1 mM pArg: 8028 molecules from 6 technical replicates

+ 0.5 mM pArg: 8642 molecules from 6 technical replicates

+ 1 mM pArg: 7393 molecules from 6 technical replicates

+ 5 mM pArg: 11494 molecules from 7 technical replicates

**Figure 3 g**

50 nM R194K: 2404 molecules from 5 technical replicates

500 nM R194K: 13148 molecules from 4 technical replicates

**Technical replicates are landing videos of the same sample.**
