# Supplementary material for: McsB forms a gated kinase chamber to mark aberrant bacterial proteins for degradation
Source: eLife. 2021 Jul 30;10:e63505. doi: 10.7554/eLife.63505 (PMC8370763; doi:10.7554/eLife.63505)
Supplement: Figure 3—source data 3. [file elife-63505-fig3-data3.docx]

**Correlation of interferometric contrast and molecular mass.**


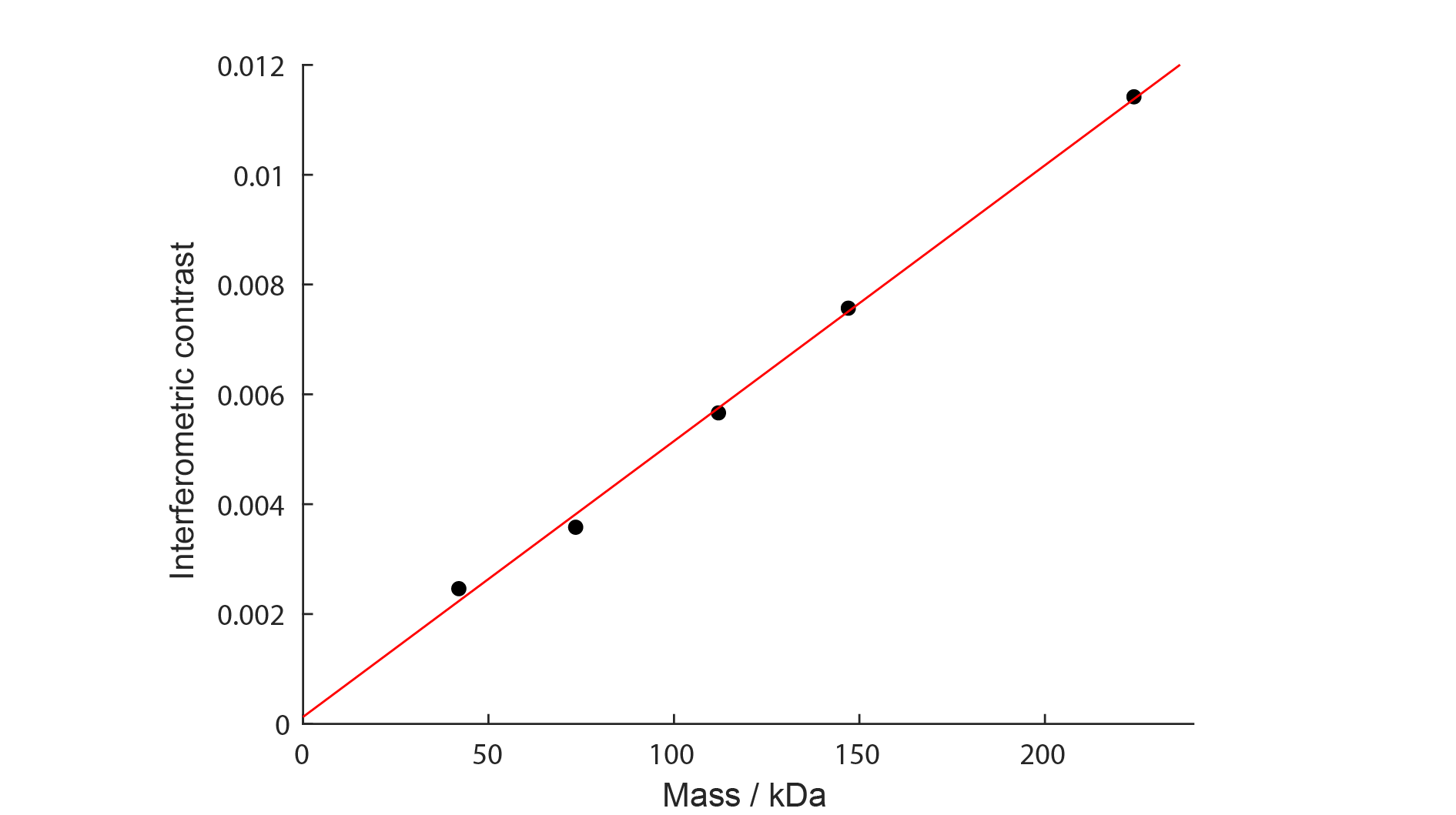


The interferometric contrast signal of landing molecules was converted into molecular mass based on the signal of standard proteins with known mass (here: protein A – 42 kDa, alcohol dehydrogenase dimer and tetramer – 73.5 and 147 kDa, β-amylase dimer and tetramer – 112 and 224 kDa).
