# Supplementary material for: McsB forms a gated kinase chamber to mark aberrant bacterial proteins for degradation
Source: eLife. 2021 Jul 30;10:e63505. doi: 10.7554/eLife.63505 (PMC8370763; doi:10.7554/eLife.63505)

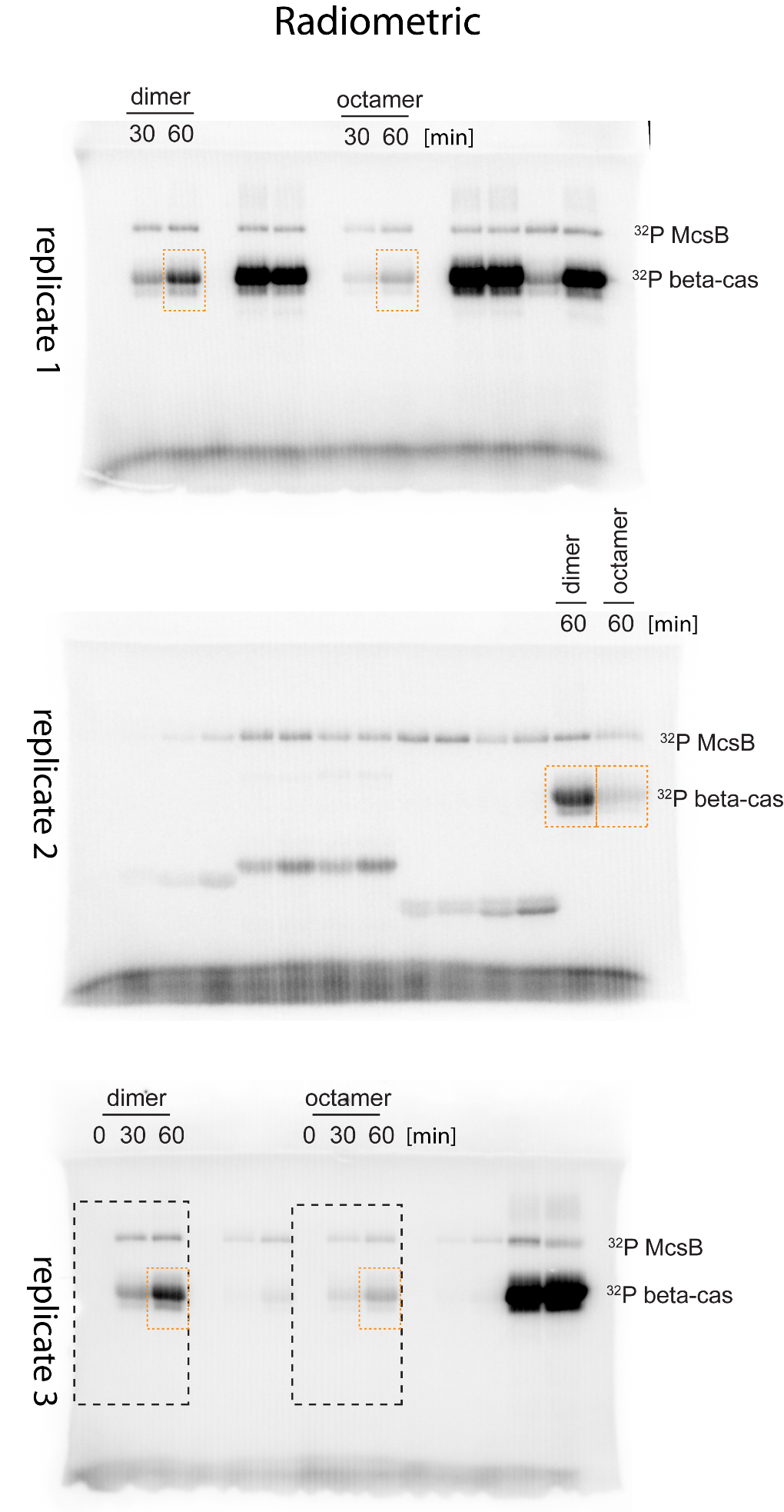
**Figure 4b**

Black boxes indicate the part shown in the main figure

Orange boxes indicate the bands used for quantification


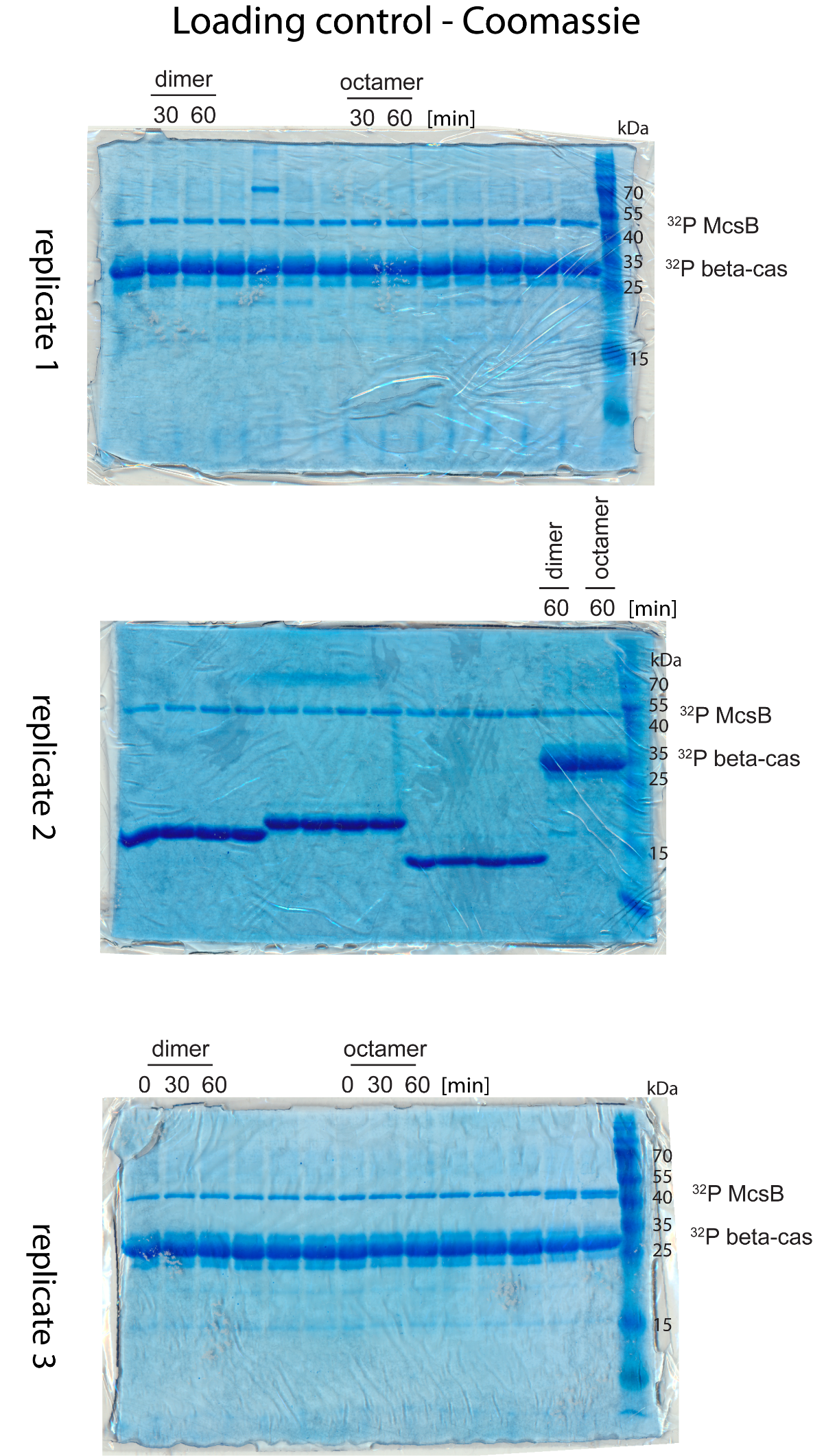


**Figure 4c**


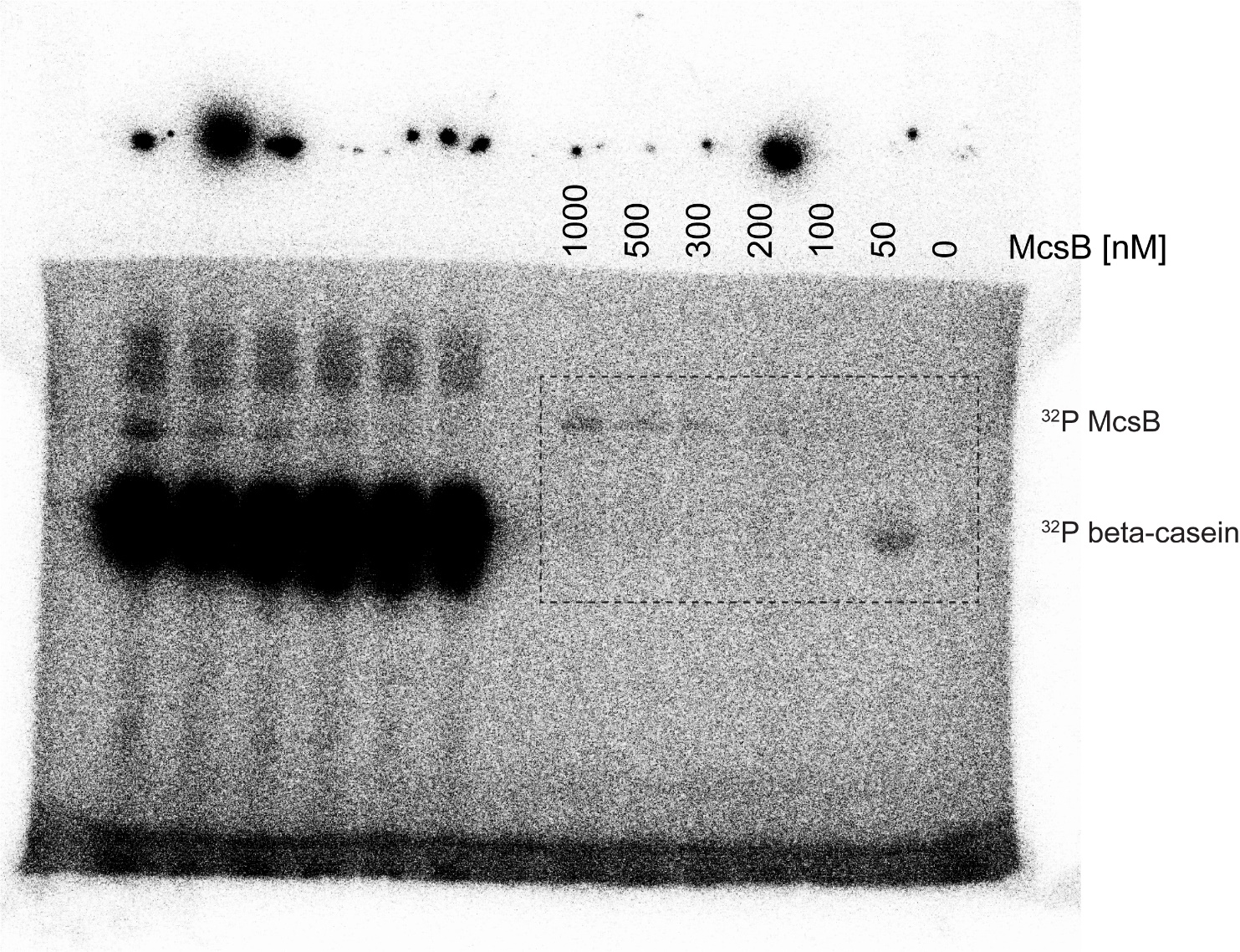

Supplement: Figure 4—source data 1. [file elife-63505-fig4-data1.docx]
