# Supplementary material for: McsB forms a gated kinase chamber to mark aberrant bacterial proteins for degradation
Source: eLife. 2021 Jul 30;10:e63505. doi: 10.7554/eLife.63505 (PMC8370763; doi:10.7554/eLife.63505)
Supplement: Figure 5—source data 1. [file elife-63505-fig5-data1.docx]

**
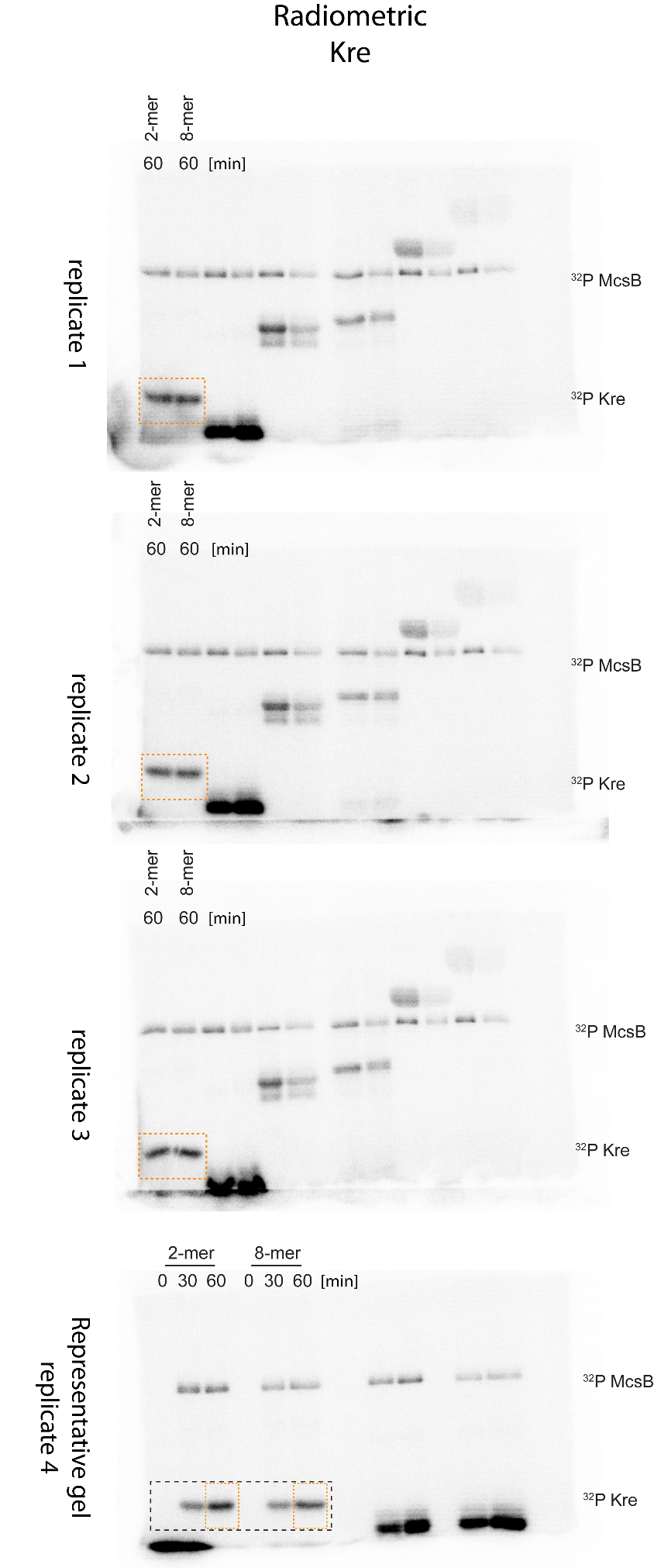
Figure 5a,b**

Black boxes indicate the part shown in the main figure

Orange boxes indicate the bands used for quantification

The contrast of each gel was adjusted individually for every substrate analyzed (over the whole gel without clipping the analyzed parts)

**
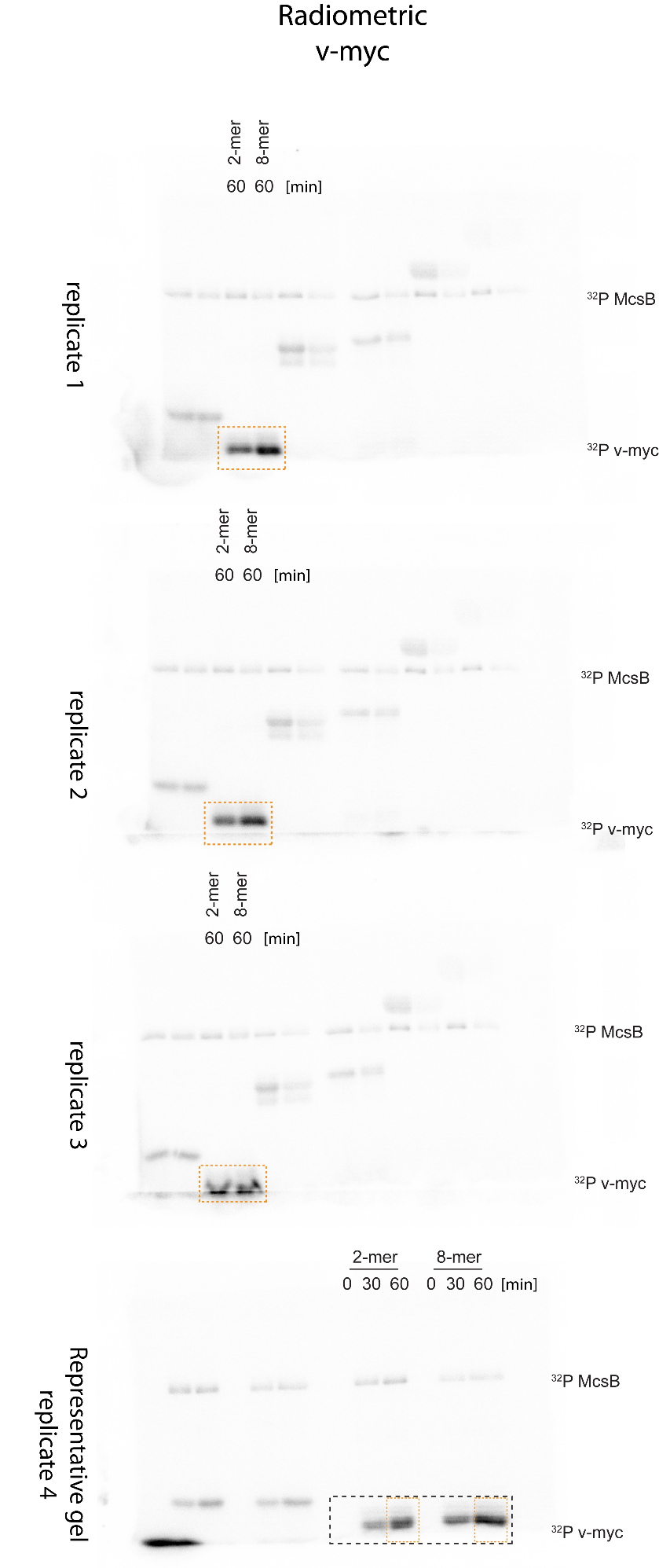
**

**
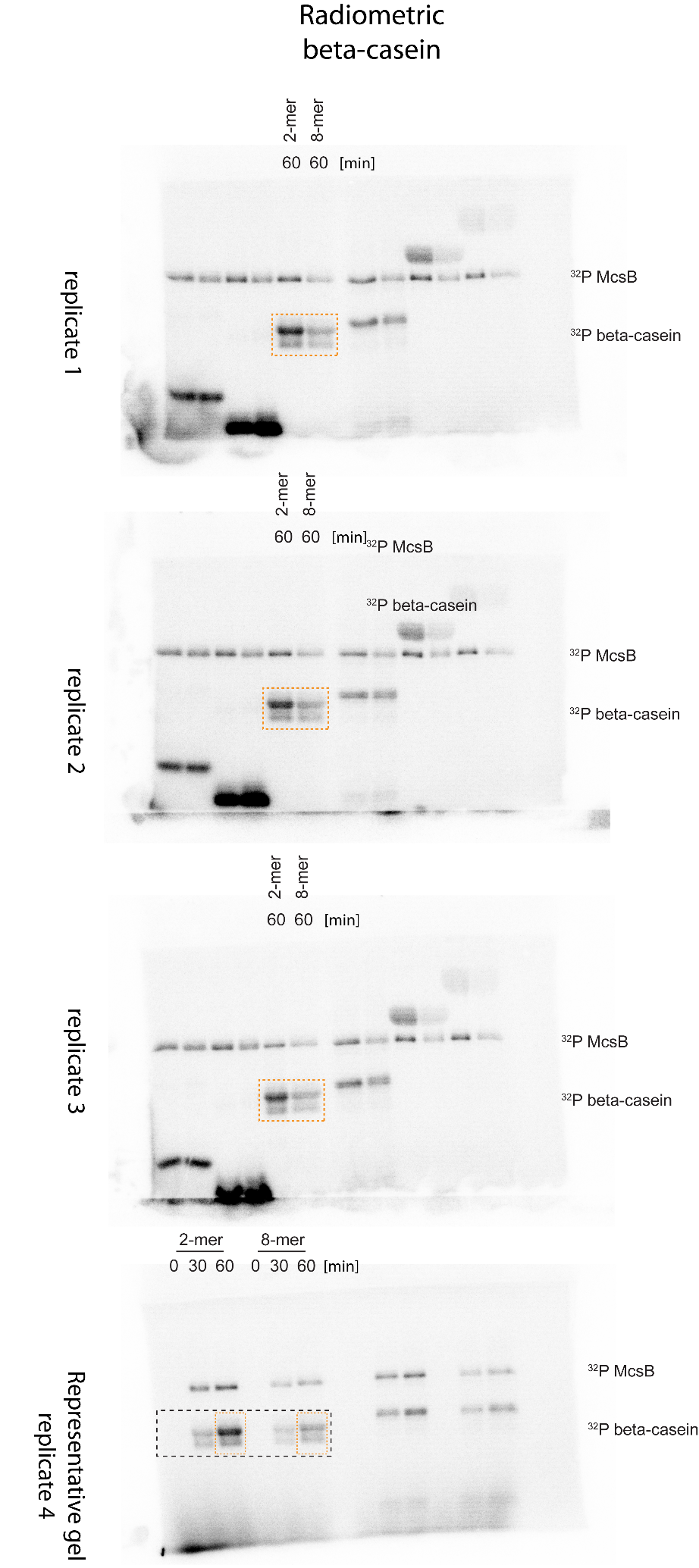
**

**
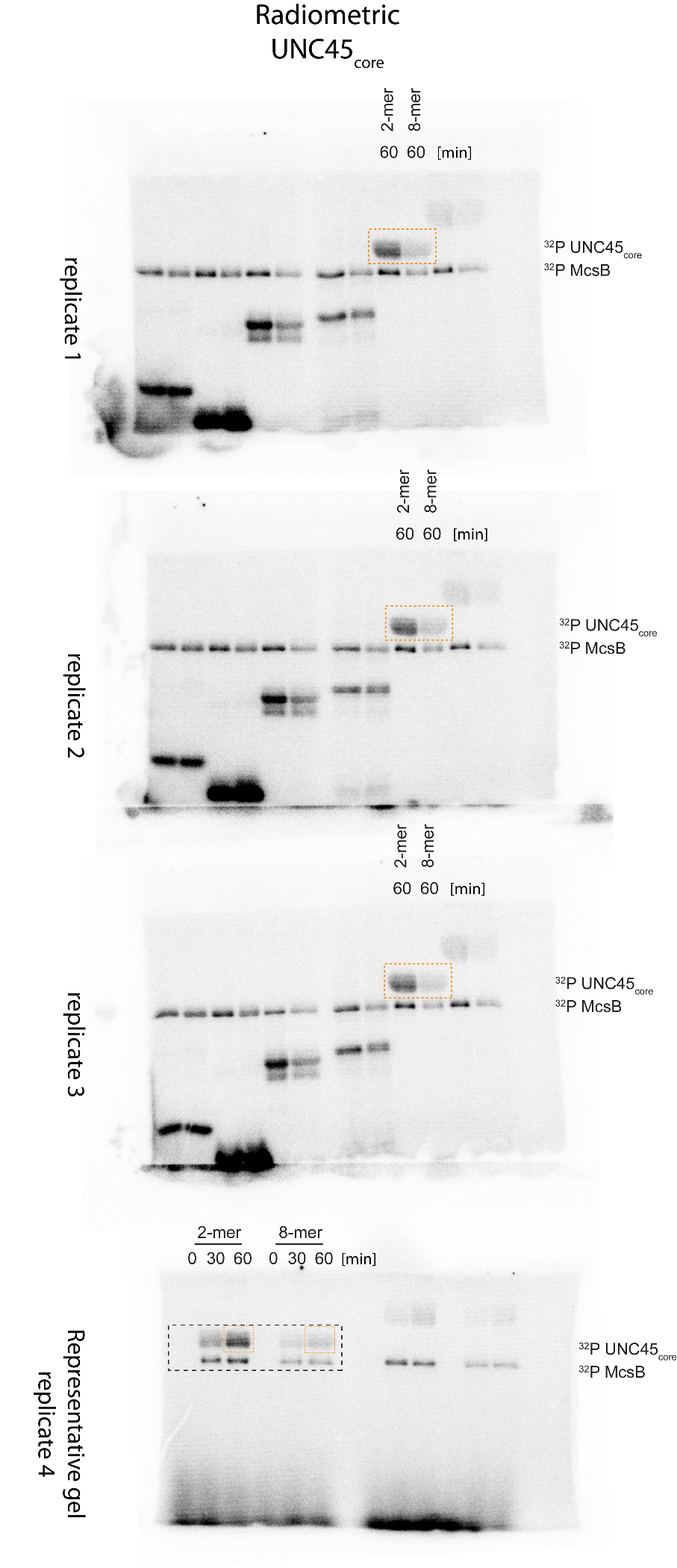
**

**
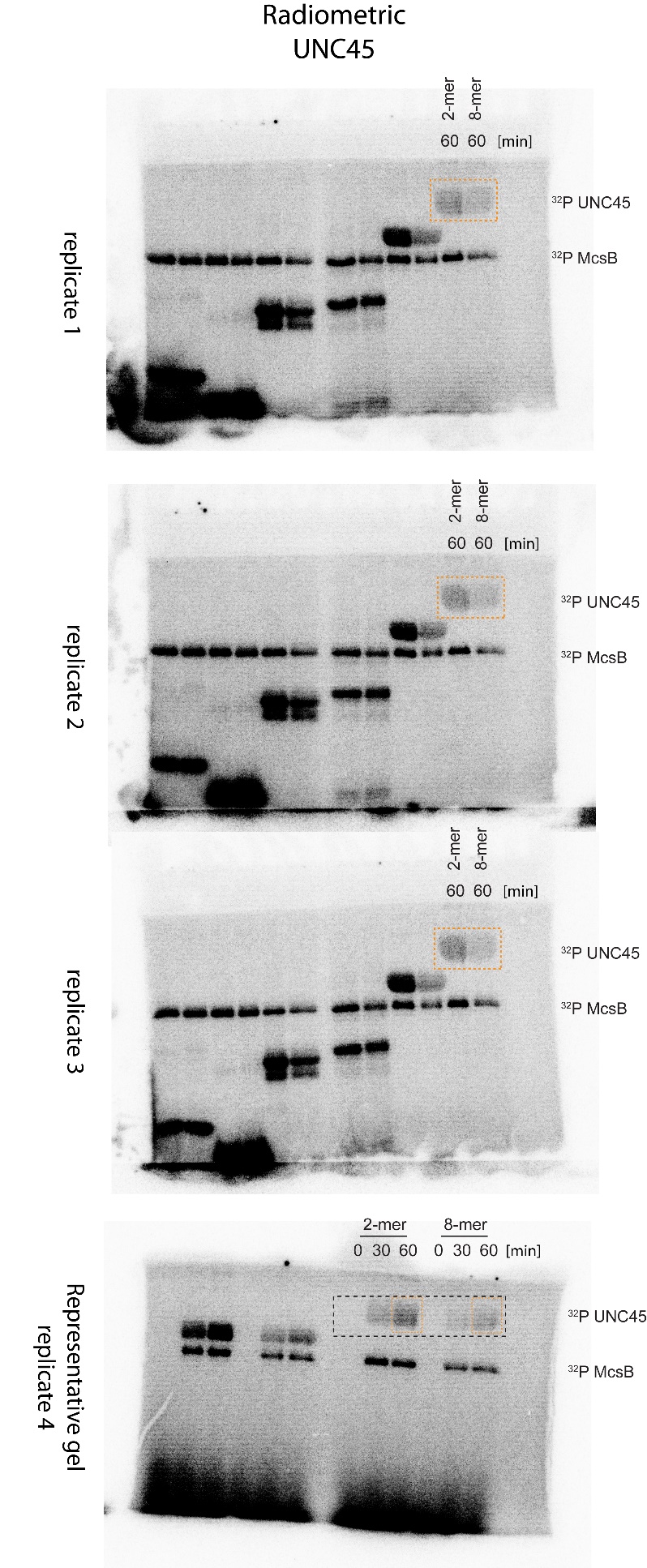
**

**
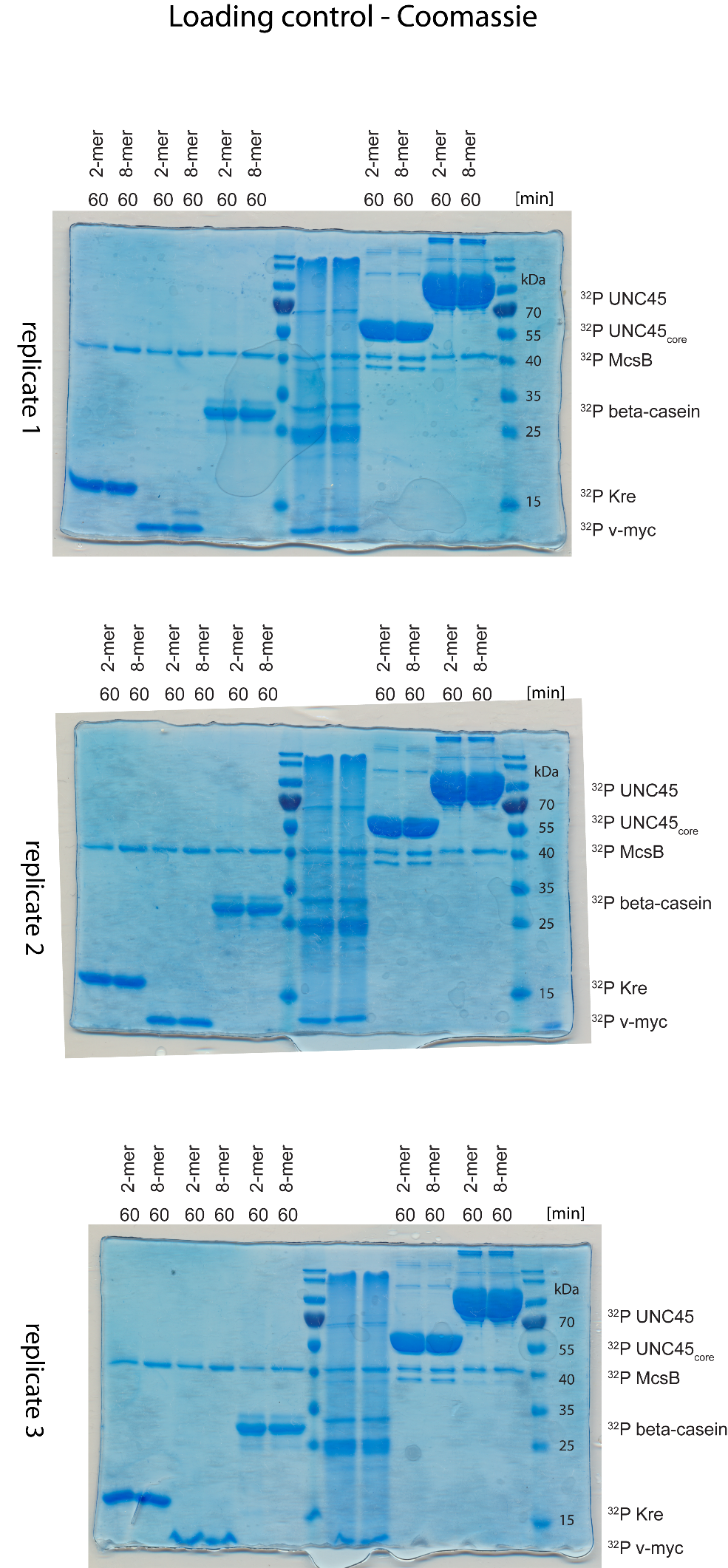
**

**
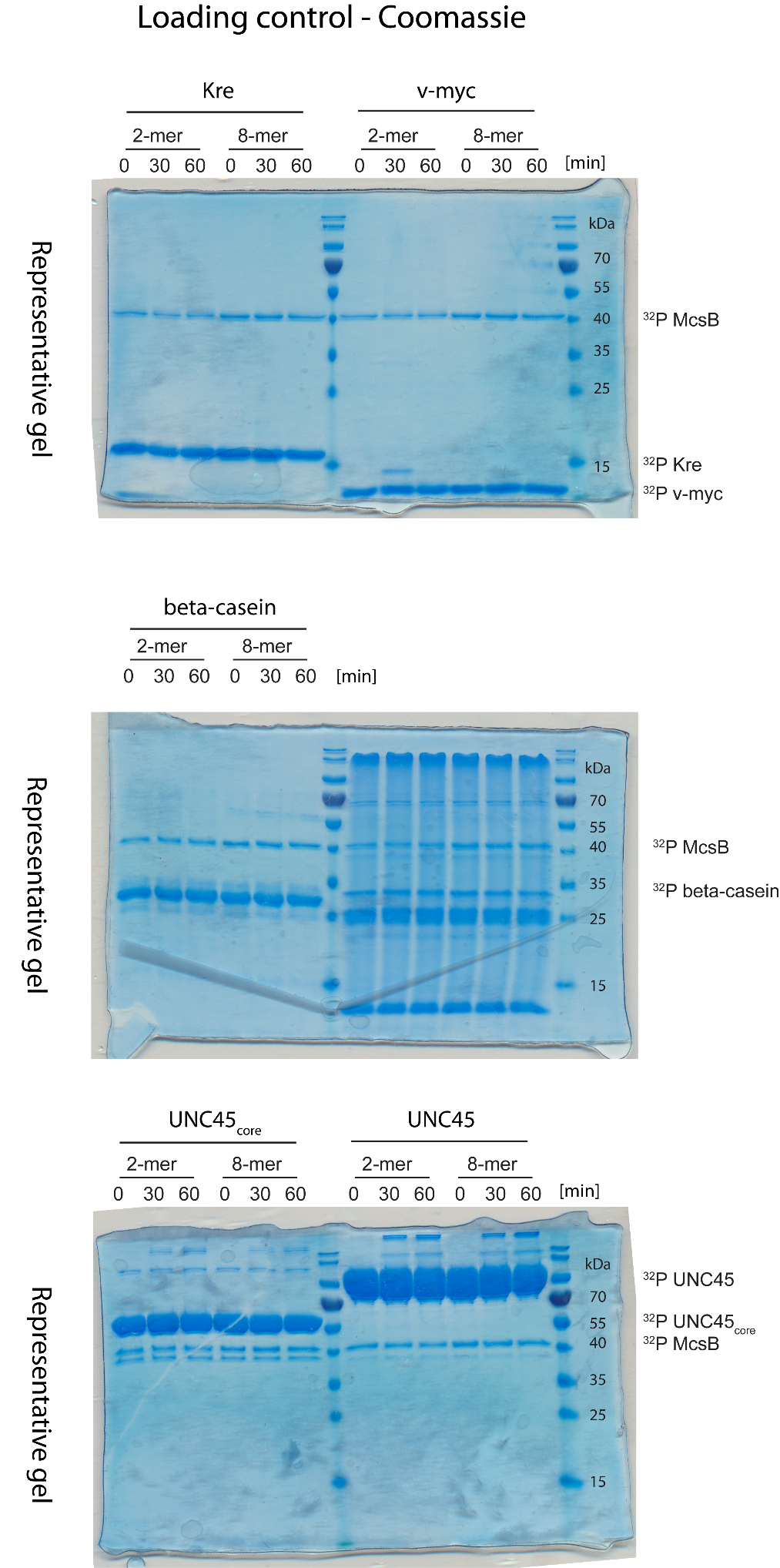
**

**Figure 5d**

Black boxes indicate the part shown in the main figure

Orange boxes indicate the bands used for quantification

**
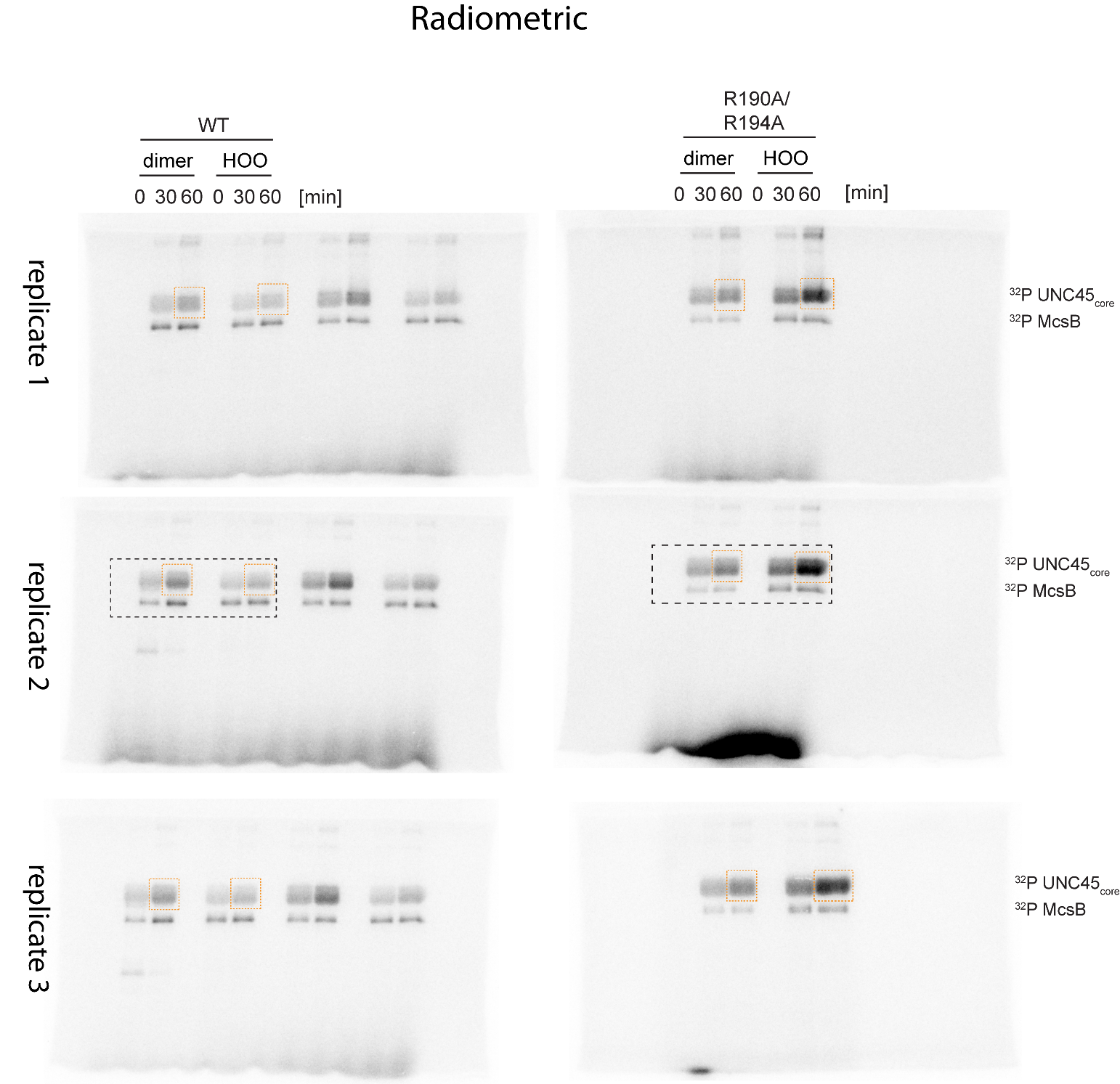
**

**
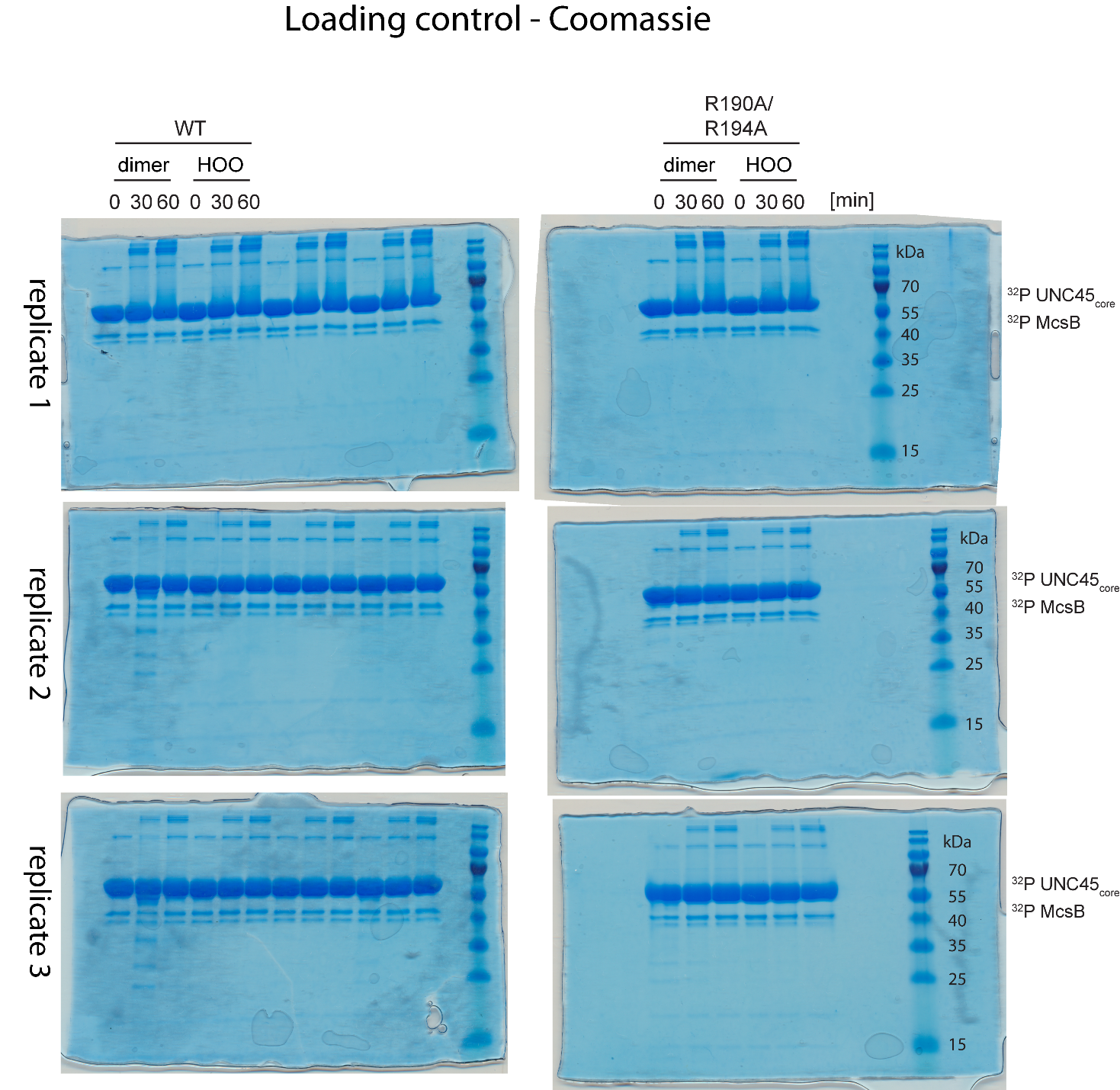
**
